# 3D Correlative Cryo-Structured Illumination Fluorescence and Soft X-ray Microscopy Elucidates Reovirus Intracellular Release Pathway

**DOI:** 10.1016/j.cell.2020.05.051

**Published:** 2020-07-23

**Authors:** Ilias Kounatidis, Megan L. Stanifer, Michael A. Phillips, Perrine Paul-Gilloteaux, Xavier Heiligenstein, Hongchang Wang, Chidinma A. Okolo, Thomas M. Fish, Matthew C. Spink, David I. Stuart, Ilan Davis, Steeve Boulant, Jonathan M. Grimes, Ian M. Dobbie, Maria Harkiolaki

**Affiliations:** 1 Diamond Light Source, Harwell Science and Innovation Campus, Didcot OX11 0DE, UK; 2 Department of Infectious Diseases, Molecular Virology, Heidelberg University Hospital, 69120 Heidelberg, Germany; 3 Micron Advanced Imaging Consortium, Department of Biochemistry, University of Oxford, South Parks Road, Oxford OX1 3QU, UK; 4 Division of Structural Biology, The Henry Wellcome Building for Genomic Medicine, Roosevelt Drive, Oxford, OX3 7BN, UK; 5 Université de Nantes, CNRS, INSERM, l’institut du thorax, Nantes, France; 6 Nantes Université, CHU Nantes, Inserm, CNRS, SFR Santé, Inserm UMS 016, CNRS UMS3556, Nantes, France; 7 CryoCapCell, 155 Boulevard de l’hôpital, 75013 Paris, France; 8 Department of Infectious Diseases, Virology, Heidelberg University Hospital, 69120 Heidelberg, Germany; 9 Research Group “Cellular polarity and viral infection,” German Cancer Research Center (DKFZ), 69120 Heidelberg, Germany

**Keywords:** correlative imaging, structured illumination microscopy, X-ray tomography, reovirus biology, CLXT

## Abstract

Imaging of biological matter across resolution scales entails the challenge of preserving the direct and unambiguous correlation of subject features from the macroscopic to the microscopic level. Here, we present a correlative imaging platform developed specifically for imaging cells in 3D under cryogenic conditions by using X-rays and visible light. Rapid cryo-preservation of biological specimens is the current gold standard in sample preparation for ultrastructural analysis in X-ray imaging. However, cryogenic fluorescence localization methods are, in their majority, diffraction-limited and fail to deliver matching resolution. We addressed this technological gap by developing an integrated, user-friendly platform for 3D correlative imaging of cells in vitreous ice by using super-resolution structured illumination microscopy in conjunction with soft X-ray tomography. The power of this approach is demonstrated by studying the process of reovirus release from intracellular vesicles during the early stages of infection and identifying intracellular virus-induced structures.

## Highlights

• Correlative cryo-imaging used to study biological processes in vitrified cells
• Reovirus intracellular release mechanism is elucidated in 3D with combined cryo-imaging
• Reovirus core particles start modifying carrier endosomes at 1 h after infection
• Correlation of X-ray and fluorescence cryo-imaging requires no data deformation

## Graphical Abstract

**Figure:**
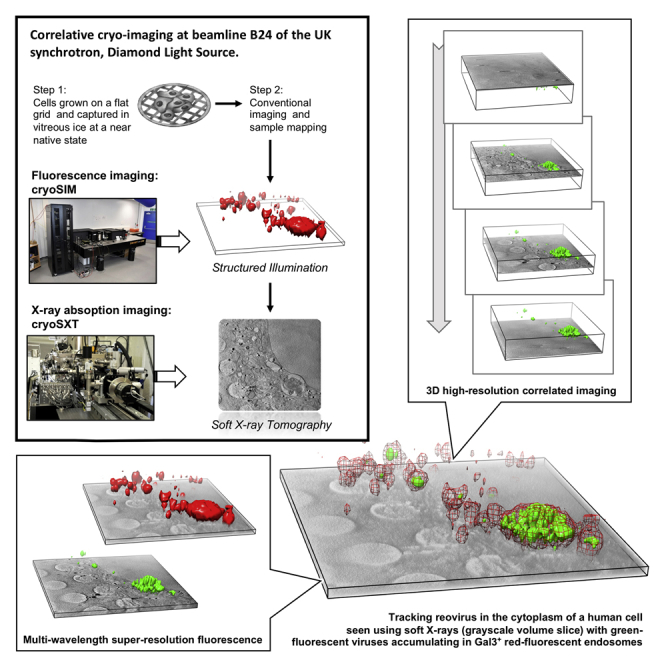

## Teaser

Kounatidis et al. describe a correlative microscopy platform that combines cryo-structured illumination fluorescence imaging with soft X-ray absorption imaging on the same sample allowing direct data correlation in 3D. The methods are used to track the intracellular progress of reovirus in human cells in the early stages of infection.

## Introduction

Cell and tissue imaging methods have developed at an impressive rate in the last decade, and new techniques have provided unprecedented opportunities for accumulating biological insights (Toomre and Bewersdorf, 2010). Currently, cellular ultrastructure can be successfully imaged to a resolution of typically 2–5 nm by using scanning or transmission electron microscopy (EM) (Zhang, 2019), although EM methods do suffer from limited penetration depth (up to 0.5 μm in transmission EM). For 3D EM visualization of thicker samples, such as mammalian cells that can easily grow beyond 3 μm in thickness, sectioning and serial reconstruction methods can be employed to overcome this limitation, but they involve chemical treatment of samples or complex and laborious cryogenic workflows (Rigort and Plitzko, 2015; Son et al., 2013). Another high-resolution 3D imaging method, soft X-ray tomography (SXT), has to date filled this need for direct mesoscale imaging of cellular ultrastructure in thicker vitrified samples, offering a penetration depth in the order of 10 μm, to a resolution of a few tens of nanometers, and the added benefit that samples require no chemical processing (Harkiolaki et al., 2018; Schneider et al., 2010). In addition, and complementary to both EM and SXT, optical microscopy continues to provide direct access to information on ultrastructure, organelle organization, and molecular localization within cells at variable resolution ranges (Giepmans et al., 2006). Recent advances in the field include methods that reach classical optical resolution limits, as defined by the Rayleigh criterion (Rayleigh, 1879), such as wide-field deconvolution (Wallace et al., 2001) and confocal laser microscopy (Sheppard and Wilson, 1981) whereas, new methods (collectively termed super-resolution microscopy) have also been developed that surpass the diffraction limit, embracing a wide range of both near-field and far-field approaches (Hell et al., 2015; Schermelleh et al., 2010). The latter have expanded rapidly over the last 20 years) leading to a number of outstanding bespoke instruments as well as off-the-shelf commercial microscopes (Betzig et al., 2006; Dobbie et al., 2011; Gustafsson et al., 2008; Hell and Wichmann, 1994; Schermelleh et al., 2008).

Beyond instrumentation, further benefits accrue in biological imaging through the combination of existing imaging methods in correlative schemes. Correlative imaging has contributed significant new insights into a range of cellular processes by providing complementary morphological, structural, and chemical information beyond what is achievable through the use of any single technique alone (Jahn et al., 2012). The process has involved correlating conventional fluorescence microscopy (FM) data with EM (Herman and Watari, 1965; Sartori et al., 2007; Schwartz et al., 2007), but recent advances have also featured super-resolution visible-light fluorescence methods (Bowler et al., 2019; Chang et al., 2014; Paez-Segala et al., 2015). In the case of SXT, correlative microscopy schemes have either incorporated diffraction-limited FM (Bai et al., 2020; Chichón et al., 2012; Duke et al., 2014; Elgass et al., 2015; Hagen et al., 2012; Le Gros et al., 2009; Meyer-Ilse et al., 2001; Myllys et al., 2016; Schneider et al., 2012; Smith et al., 2014) or involved a chemical fixation step to take advantage of super-resolution FM (Varsano et al., 2016) with the associated risk for artifact formation (Li et al., 2017; Pereira et al., 2019).

One of the challenges of high-resolution imaging of cells and tissue has been the preservation of native cellular structures and organization. For that reason, as an alternative to the widely used practice of chemical fixation, rapid cryo-freezing has emerged as the current gold standard for preserving delicate biological structures or arresting rapid dynamic processes in cells (Echlin, 1991; Koning et al., 2018) followed by cryo-imaging (Hell et al., 2015). Vitrification was identified as a promising technique for biological sample preservation for ultrastructural imaging 40 years ago (Dubochet and McDowall, 1981) and since then has developed into the method of choice for sample preparation for both X-ray imaging (Duke et al., 2014) and electron tomography (Koning et al., 2018) and has also been used for super-resolution fluorescence imaging (Hoffman et al., 2020; Kaufmann et al., 2014). Vitrified samples suffer no fixation artifacts and cellular structures are preserved faithfully in their pre-vitrification state (Kaufmann et al., 2014). Fortuitously, fluorescence imaging at cryogenic temperatures is characterized by enhanced photo-stability of dyes and sharper emission spectra as compared to room temperature imaging leading to data with better signal to noise ratios (Kaufmann et al., 2014; Moerner and Orrit, 1999; Schwartz et al., 2007). However, the numerical aperture (NA) in cryo-FM systems is limited because there are currently neither suitable immersion fluids nor robust dipping objectives. As a result, cryo-fluorescence imaging is done by using air objectives with a maximum NA of circa 0.9, limiting the achievable optical resolution, and it still remains technically challenging to prevent ice crystal formation leading to sample damage while imaging under cryogenic conditions.

Given these challenges, 3D-structure illumination microscopy (3D-SIM) emerges as the method of choice for imaging under cryogenic conditions (Demmerle et al., 2017; Gustafsson, 2000; Gustafsson et al., 2008; Heintzmann and Cremer, 1999; Karadaglić and Wilson, 2008). Structured illumination microscopy (SIM) involves the use of patterned light that interacts with fluorophores on the imaging target resulting in low-frequency interference fringes (Moiré effect) that carry structural information beyond the diffraction limit of the system and can lead to an 8-fold increase in volumetric resolution. SIM has many distinct “selling-points” for cryogenic imaging: (1) it is easily applied to multi-channel imaging by using a range of common dyes or fluorescent proteins, opening up a wide range of biology for study; (2) it can image relatively thick samples, reducing the need for sample sectioning prior to imaging; (3) it uses relatively low light doses (10–100 W/cm^2^ laser power) and short image acquisition times (20–100 ms per single exposure), minimizing sample heating and, (4) it has extremely good out of focus light suppression, leading to high contrast images even in samples with thicknesses of 10 μm or more (Demmerle et al., 2017). Indeed, SIM has been recently employed within a correlative scheme alongside single molecule localization microscopy to visualize cells in vitreous ice before imaging with scanning EM via focused-ion beam milling on the same cells after they are freeze-substitution stained (Hoffman et al., 2020).

Here, we present a correlative imaging scheme for biological samples at cryogenic temperatures that uses a purpose-built 3D-cryoSIM and a synchrotron soft X-ray microscope capable of tomographic imaging. These are available as an accessible and user-friendly imaging platform at beamline B24 (Harkiolaki et al., 2018) at the national UK synchrotron, Diamond Light Source (DLS) (https://www.diamond.ac.uk). The techniques implemented are highly complementary and deliver comprehensive views of both cellular ultrastructure and molecular organization over extended cellular volumes in minimally perturbed cell populations. Moreover, once samples are vitrified, no further treatment or modification is required to go from one imaging modality to the next, and whole cell populations can be imaged at different microscopes in a matter of hours. We have applied this combination of imaging technology toward the investigation of the early events of reovirus infection in mammalian cells and, in particular, the mechanism of viral penetration of endocytic vesicles. Reovirus is an established experimental model for viral pathogenesis and has potential applications in the development of anticancer treatments (Roy, 2006; Thirukkumaran and Morris, 2009).

Viruses are infectious particles, which must enter a host cell to propagate. Therefore, crucial to any viral infection is the passage through the physical barrier of host membranes. For enveloped viruses, the mechanisms of virion delivery are relatively well understood and rely on fusion of the lipid bilayer of the viral envelope with the lipid bilayer of the host (White and Whittaker, 2016). Reovirus, however, as a non-enveloped icosahedral virus, does not have membrane fusing capacity, and how its core reaches the cytoplasm to initiate replication has remained largely elusive to date. Reoviruses have a segmented double-stranded RNA genome, which during viral infection is retained within the closed core particle, preventing the triggering of cellular innate immune responses. Viral core particles have both the RNA polymerase and the capping enzyme required for transcription of viral mRNA and viral replication. The core particle of reovirus is cloaked by the outer capsid, which is composed of proteins σ3 and μ1, plus the σ1 spike protein at the icosahedral 5-fold axes. σ1 and σ3 are involved in receptor attachment whereas μ1 plays a role in viral entry (Zhang et al., 2006) Reoviruses have been shown to enter cells via clathrin-mediated endocytosis and are subsequently trafficked through the endosomal pathway from the early Rab4-, Rab5-, or Rab11-positive endosomes to the late Rab7- or Rab9-positive endosomes (Boulant et al., 2013; Mainou and Dermody, 2012). The escape of reovirus core particles from these endosomes to the cytoplasm has been linked to low-pH-dependent proteolytic degradation events (Dermody et al., 1993). There is *in vitro* evidence that proteolytic removal of σ3 (likely to occur in endosomes because of gradual increase in acidity and digestion by low-pH dependent-cathepsins) (Ebert et al., 2002), brings about the release of the N-terminally myristoylated μ1 peptide, which leads to pore formation (Sturzenbecker et al., 1987). Important unanswered questions regarding the mechanisms driving the early stages of reovirus infection include: (1) how soon after entry are replication-competent core particles released into the cytoplasm, (2) are they released at specific locations within the cytosol, and (3) is the ultrastructure of endosome vesicles affected by viruses during their entry, trafficking, and release? Our current lack of understanding of the details of core particle intracellular release is, in large part, due to the absence of technological solutions to visualize, at sufficient resolution, the cellular landscape during infection and its remodeling in response to viral infection.

## Results

### Design and performance of the 3D cryo-structured illumination microscope (CryoSIM)

Given the current requirement to extend the application of super-resolution methods to the study of cryogenically preserved samples for the purposes of correlative imaging, we present here a 3D-SIM setup with an open optical design that overcomes many of the technical difficulties of 3D imaging fluorescence at super-resolution under cryogenic conditions. The conceptual starting point of reference for our design was the OMX platform (Carlton et al., 2010; Dobbie et al., 2011; Gustafsson et al., 2008; Schermelleh et al., 2008); detailed design and implementation parameters for both optics and software can be found at Dobbie et al. (2020).

**Figure 1:**
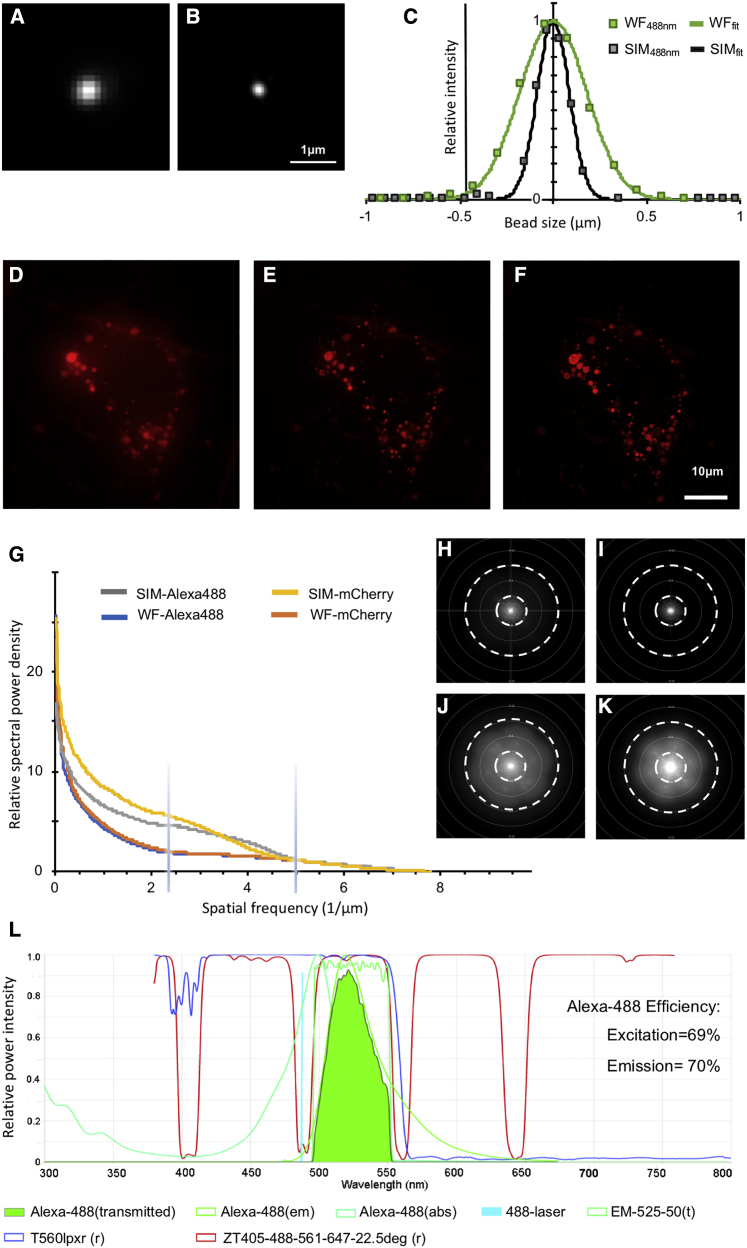
Design and performance of the super-resolution fluorescence microscope CryoSIM (A and B) Widefield (WF) (A) and SIM lateral point spread (B) of a 175 nm diameter, 505/515 nm wavelength microsphere (PS-Speck Thermo Fischer Scientific) collected on the cryoSIM at 71 K by using 488 nm light for excitation and a 520/35 nm filter (Semrock) for emission. (C) Plot of lateral point spread (single line profile) of (A) and (B) and their respective Gaussian fits showing clear resolution enhancement. Analysis of representative cryo-SIM data from a single mammalian U2OS cell containing structures tagged with mCherry. (D) Z axis projection of the raw SIM data processed to produce a widefield image stack. (E) Z axis projection of the same data deconvolved with a standard Richardson-Lucy iterative deconvolution algorithm. (F) SIM data fully reconstructed showing the resulting resolution enhancement. (G) Fourier information content analysis of (H)–(K) (flattened and sampled radially) showing the relative increase in information content after reconstruction. The vertical blue bars show the resolution achieved at 525 nm emission in widefield (420 nm, 1/2.38) and SIM (200 nm, 1/5). (H and I) Reciprocal space resolution plots of the widefield data in the cell shown in (D) (Alexa488 and mCherry signal, respectively). (J and K) Same data as (H) and (I), respectively, from the SIM reconstruction shown in (F). Comparing (J) with (H) and (K) with (I) shows the increased image resolution in SIM by the extra intensity further from the origin and therefore at higher frequencies. Concentric dashed circles denote resolution boundaries of 600 nm (small circle) and 200 nm (large circle). All image analysis was done using Fiji (Schindelin et al., 2012) and SIMcheck (Ball et al., 2015). (L) SPEKcheck (Phillips et al., 2018) display of the cryoSIM for a representative fluorophore (Alexa488) excited in the system with an efficiency of 69% and a resulting collection of 70% of the total emitted light. Laser light is shown as a bright blue spike, excitation and emission are shown in shades of light green, and the total transmitted light is shown in bright green. The system dichroic mirrors and filters are also represented (Abbreviations are as follows: ZT, system dichroics; T560, detector splitter dichroic; EM, emission filter).

**Figure S1:**
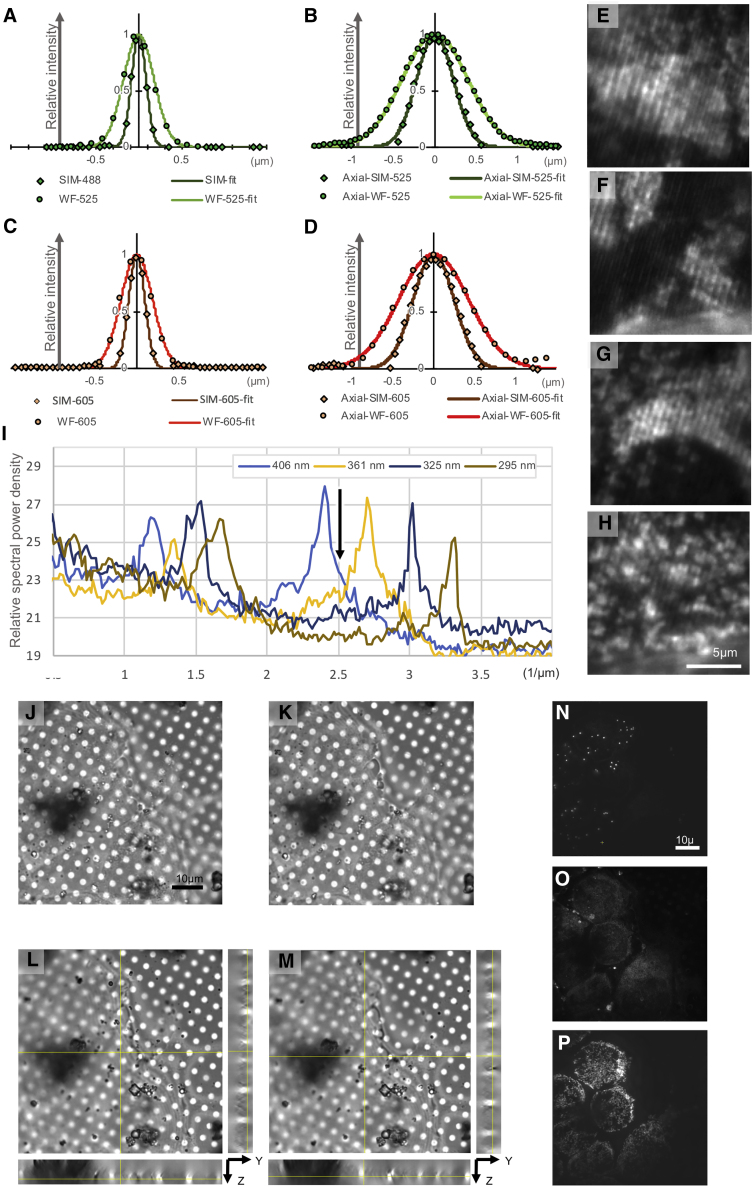
CryoSIM resolution parameters, related to Figure 1 Line scans laterally (A and C) and axially (B and D) through single fluorescent beads at 525 nm (A and B) and 605 nm (C and D) emission in widefield and SIM images. Widefield data presented as circles and SIM as diamonds with Gaussians fits as lines. In (E–H) are parts of a raw SIM image of fluorescent mitochondria with decreasing stripe width. 2nd order stripes are 406, 361, 325 and 295 nm respectively. In (I) are line scans through Fourier transforms of the data in (E–H), with varying SIM illumination stripe widths, showing the peaks due to the first order stripes (around 1–1.8 μm^−1^) and second order (around 2.4 to 3.5 μm^−1^). As stripe width decreases, second order peaks decrease in amplitude and shift along the y axis. The black arrow indicates the stripe width, 396 nm, used for the experimental data in the paper. Representative brightfield image of vitrified U2OS cells (J), before (pre-exposure) and (K), after (post-exposure) seven cycles of cryoSIM imaging of green, red, and far red intracellular fluorophores (525 nm, 605 nm and 647 nm) showing that there is no localized ‘melting’ due to exposure to laser light for SIM purposes. In (L) and (M), are orthoslices of (J) and (K), respectively. SIM imaging from the last round of data collection shows the preserved cellular features: (N), green fluorescent lipid droplets, (O), red fluorescent endoplasmic reticulum and (P), far-red fluorescent mitochondria corresponding to the brightfield data shown in (K).

This custom-designed cryoSIM is built at beamline B24 around a commercial cryo-stage (CMS196M, Linkam Scientific) with a long working distance air objective (100×, 0.9 NA, 2 mm working distance, Nikon) and delivers imaging at resolutions beyond the diffraction limit (see Figures 1A–1K, S1A–S1D, and Table S1 for optical performance with nanobeads and cells). By using a relatively high NA long-working-distance air objective, we are able to maintain samples at cryogenic conditions (71 K) without dipping the objective in cryogen. The system currently has four illumination wavelengths (405, 488, 561, and 647 nm) from individual direct-diode and diode-pumped solid-state lasers, which could, if necessary, be modified to include any other separable channels in the visible wavelength range. A nematic liquid crystal spatial light modulator is used in phase modulation mode to produce structured illumination (SI) stripe patterns, and a liquid crystal based polarization rotator (Meadowlark Optics) allows us to optimize SI patterns independently for each stripe orientation and excitation wavelength, eliminating the need to optimize for one to the detriment of all others (see Figures S1E–S1I and STAR Methods for further information on the working optical setup). The system’s optical features are incorporated under the label “cryoSIM” in the available-online Spekcheck tool (Phillips et al., 2018) where the efficiency and performance of different fluorophores can be assessed *in silico* with respect to features in our setup, thereby enabling the intelligent design of fluorescence-dependent imaging experiments (Figure 1l). 3D-SIM requires 30 times as many images as widefield fluorescence, 15 images per z-plane, and twice as many z-planes to satisfy Nyquist-Shannon sampling (Shannon, 1949). At the cryoSIM, each z slice is recorded 15 times (five phases for each of three angles in the SI pattern) at 125 nm increments along the z axis as 512 × 512 pixel images with 125 nm per pixel and reconstructed to 1,024 × 1,024 pixel image stacks with voxel size of 62.5 × 62.5 × 125 nm. Each exposure depends on the overall fluorophore intensity, but commonly we expose for tens of milliseconds using laser light at 10–100 mW/cm^2^ illumination power. Because the sample remains cryo-protected at all times during data collection, devitrification or crystalline ice formation is avoided (Figures S1J–S1P). A typical dataset is collected within an average time of 3–5 min capturing a field of view (FOV) of 64 × 64 μm in all z slices required. Point spread functions for reconstruction purposes at cryogenic temperatures are generated before data collection using 175 nm *PS-Speck* nanobeads (Thermo-Fischer Scientific) at emission wavelengths of 440, 525, 605, and 647 nm.

### Beamline and X-ray microscopy setup for Soft X-ray tomography (Cryo-SXT)

**Figure 2:**
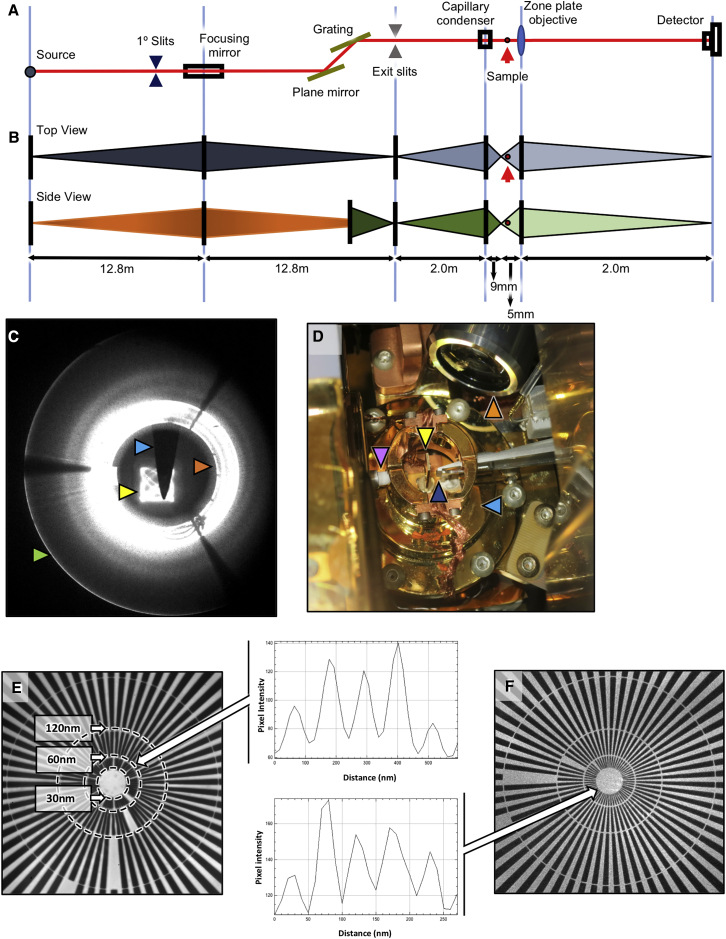
TXM optical features and resolution attained (A) Schematic representation of the X-ray optical path at beamline B24. (B) Beam divergence profile and monochromacy, top and side view. The bending magnetic source light is focused by horizontal deflection of 1.1° with 1:1 magnification by a toroid focusing mirror located 12.8 m downstream. A plane mirror-grating monochromator directs the real image of the source light, now monochromatic, a total of 25.6 m from the bending magnet it originated from. This beam is then focused by a capillary condenser to the sample and a zone plate objective delivers the magnified image to the detector. (C) Imaging beam profile recorded at a scintillator 0.5 m downstream of sample position; the focused beam is used to fill a central square FOV (yellow arrow) with a Lissajous light pattern the result of a two-motor condenser sweep, with light reflected from the condenser at the maximum angle (green arrow) and the inner surface of the condenser (red arrow). An alignment Tungsten pin (blue arrow) can be seen at the sample position in the middle of the illuminated area. (D) View of the sample environment in the TXM showing the condenser (purple arrow), the objective (dark blue arrow), the sample holder (yellow arrow), the 20× visible-light objective (orange arrow) and the cryo-shield (light blue arrow; needed to minimize thermal drift between the 71 K “cold” sample and the optics that are all maintained at 21°C; sample chamber maintained at high vacuum <10^−6^Torr). (E and F) X-ray projection of a 30 nm (minimum spacing) Siemens star at beamline B24 imaged with 40 nm and 25 nm objectives, respectively (optical resolution for each zone plate is documented through the imaging of individual spokes at progressively higher resolution zones on the star pattern); resolution is measured by observable dips in absorption at >2 sigma by using a line profile across several spokes at the inner edge of each concentric zone. Arrows emanating from the associated graphs show the resolution edge the line profile was collected. Dotted circles denote the edge of each zone and the spacing therein. Line profiles were generated with Fiji (Schindelin et al., 2012); vertical axis shows pixel intensity values; The TXM delivers resolution beyond 60 nm and 30 nm resolution with the 40 nm and 25 nm zone plate objectives, respectively.

The full field transmission X-ray microscope (TXM) (UltraXRM-S220C, Carl Zeiss X-ray Microscopy Inc.) is a synchrotron endstation installed at beamline B24 (DLS, zone 4), which delivers imaging by absorption contrast (Figures 2A–2D). The TXM uses as illumination a defined spectral region of the X-ray spectrum known as the “water window,” which lies between the k absorption edges of carbon at 284 eV and oxygen at 543 eV. Within this region, carbon-rich biological structures absorb X-rays more than the surrounding oxygen-rich medium, and the resulting natural contrast allows the delineation of cellular features in recorded projections. Cellular membranes provide particularly strong contrast, making membrane bounded organelles especially easy to identify within relatively thick ultrastructural 3D tomograms of whole cells.

**Figure S2:**
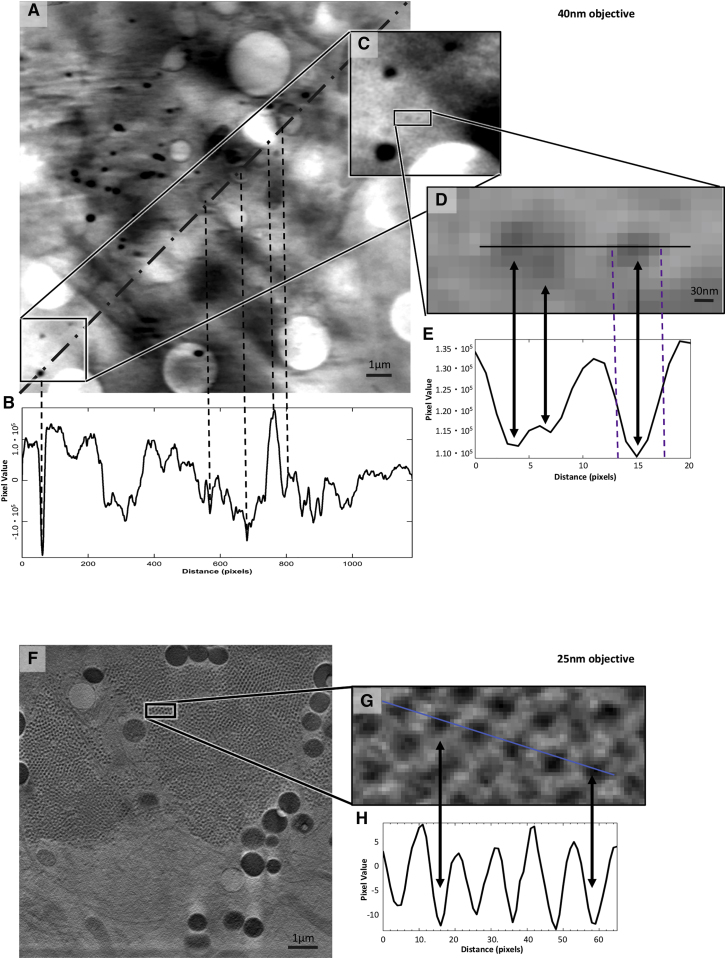
X-ray absorption data contrast in cells using different TXM objectives, related to Figure 2 (A) X-ray projection of a 16x16 μm FOV collected at the beamline B24 TXM using the 40 nm objective in a U2OS mammalian cell sample 1h after it has been exposed to infectious reovirus at MOI of 50. The zoomed view in square at the bottom left of the image is located outside the cell. The backing surface (Quantifoil™) can be identified by the regular pattern of circles (needed for blotting prior to vitrification). (B) The diagonal dotted line defines the line used to generate (B), a line profile of pixel intensity in Fiji (Schindelin et al., 2012). Dips in the profile correspond to features of high X-ray absorbance such as membranes and organelles (corresponding vertical dotted lines have been added as examples). We expect reovirus particles to be present both in and outside the cell but given the high background it is be difficult to unambiguously identify. (C and D) Closeups of marked areas showing contrast that could be attributed to reovirus particles. (E) The associated line profile, clearly contains peaks that are 4-5 pixels apart; at 16nm per pixel that gives an object diameter of approximately 80 nm which matches the expected diameter of a reovirus particle. However, the ratio of pixel values is at best 1.1:1.3 even in this ‘ideal’ area (low background without any overlap with cellular content) making the unambiguous identification of individual virus impossible in this sample. (F) X-ray projection of a 10x10 μm FOV collected at the B24 TXM using the 25 nm objective in the cytoplasm of a BSC-1 cell 16 h after it has been exposed to infectious reovirus at MOI of 50-100. The observed regular pattern of small dots is indicative of viral factories with each of these dots representing a single viral particle. (G) A close up of the boxed area in (F), with a line drawn to generate (H), a line profile of pixel intensities. Full width half maxima of this plot give an average diameter of 60nm for these particles which appear to be an array of immature particles in the viral factory having yet to assemble an outer capsid before cell exit.

**Figure 3:**
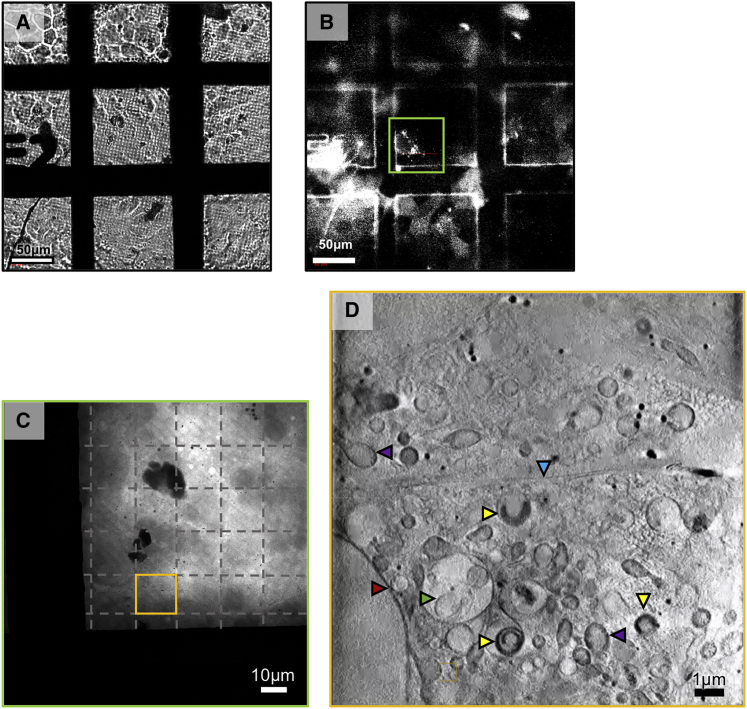
Visible light mapping and X-ray imaging process at the TXM (A and B) Brightfield (A) and fluorescence (B) in line visible-light imaging of a sample grid supporting a mixed fluorescence cell population. (C) 2D X-ray mosaic in the ROI denoted in (B) (green outline with individual FOVs delineated by hashed lines). (D) Middle slice from the X-ray tomogram collected in the ROI denoted in (C) (orange outline). Characteristic cellular features are identified by arrows: red for nuclear envelope, purple for mitochondria, green for MVBs, blue for the contact area between two adjacent cells, and yellow for reovirus-carbon-rich structures within vesicles.

At B24, there is a choice between a 25 nm and a 40 nm resolution setup for absorption contrast imaging (depth of focus of circa 1 and 2.6 μm, respectively) (Figures 2E and 2F; Table S2). Although the higher resolution setup provides for a more detailed view of biological structures (especially pertinent in the study of finer features and pathogens), the lower resolution delivered by a 40 nm zone plate objective is leveraged by increased FOV and depth of focus making the latter best suited for studies aimed at collecting the maximum amount of ultracellular structure, such as the study of organelle distribution, shuttling, and restructuring. The TXM delivers magnification up to 1,300× with a square FOV of view of between 10 μm or 16 μm across and records 1,024 × 1,024 pixel projections at either 10 nm or 16 nm per pixel, depending on the objective in use (25 nm and 40 nm, respectively) (see Figure S2 and Table S2 for further analysis of resolution parameters in cryoSXT). In addition, it provides basic in-line visible light microscopy capacity with broad-spectrum LED illumination (widefield and fluorescence) through a 20× objective located within the sample chamber (Figure 3) to allow mapping and registration of regions of interest (ROIs) previously identified by conventional or super-resolution light microscopies (see below).

For non-destructive SXT imaging at beamline B24, samples need to be embedded in vitreous ice and mounted on flat EM grids to immobilize them and preserve them against radiation and heat damage before exposure to X-ray radiation. Gold nanospheres, 100–250 nm in diameter, are traditionally included in sample preparation just before vitrification to provide highly absorbent points of reference (fiducials) for image alignment and subsequent data reconstruction. 3D imaging data are collected as a series of projections at defined regular angular steps generating tilt series of up to 140° in total with projections collected typically with 0.5–1 s X-ray exposures per image. The images are subsequently processed into tomograms by using IMOD (Kremer et al., 1996) through a fully automated, *in silico* pipeline that runs concurrently with data collection. To capture cellular content extending beyond a single FOV, multiple tomograms can be collected at adjacent overlapping areas (e.g., when vesicles and substructures of interest need to be captured along with the overall cellular landscape in which they reside) (Harkiolaki et al., 2018).

It is important to note that deciding on a data collection strategy before acquisition has proven a vital step for subsequent correlative work. Parameters such as sample thickness and susceptibility to radiation damage (heating due to X-ray absorption) need to be balanced against optimal single frame exposure time and sampling step for the resolution required, whereas sampling of multiple overlapping FOVs is entirely dependent on how robust the sample is to extended exposures. Moreover, background noise is accumulated in the raw data images because of sample holder materials such as thin carbon support film, soluble intracellular content, or surrounding leftover cell media in combination with sample or beam mechanical instabilities during data collection that can reduce contrast in the data and interfere with the delivery of the best achievable resolution (Figures 2E, 2F, and S2).

### Experimental workflow of correlative imaging using Cryo-SXT and Cryo-SIM

**Figure 4:**
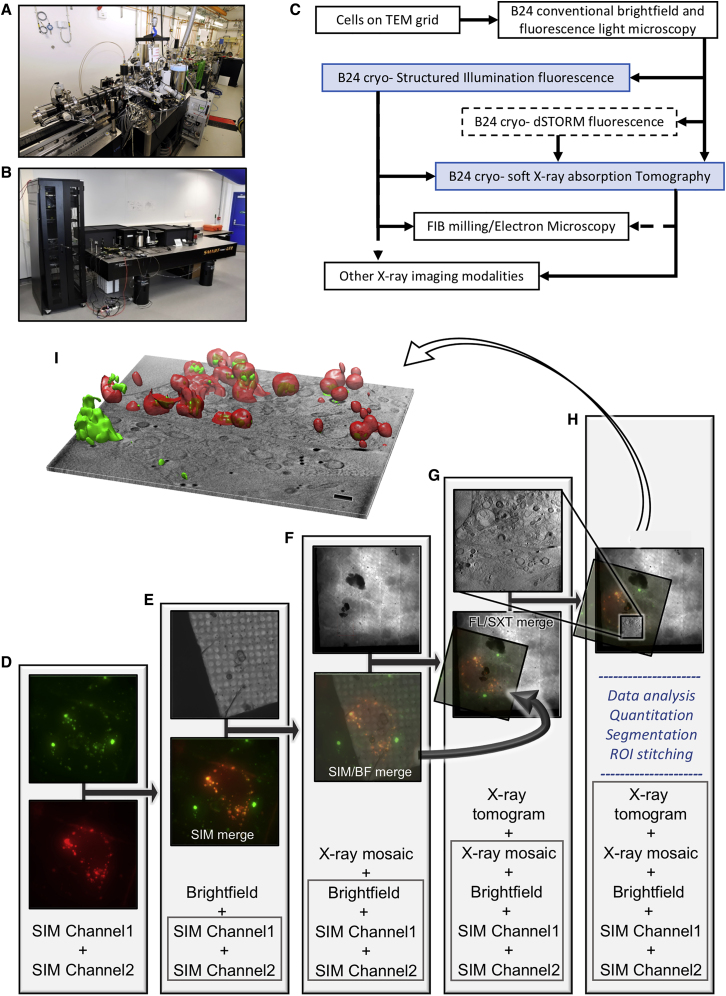
Correlative imaging tools and workflow at beamline B24 with stepwise protocol for *in silico* correlation of data collected by using the different microscopes (A and B) Images of the TXM (A) and the cryoSIM (B) on site at the Diamond Light Source beamline B24 (https://www.diamond.ac.uk/Instruments/Biological-Cryo-Imaging/B24.html). (C) A schematic of the operational workflow that incorporates cryoSIM and cryoSXT as well as other imaging applications and potential imaging partnerships (hashed lines denote methods and correlative paths currently under development). (D) Correlation of fluorescence signals on the same ROI. (E) Superposition of all fluorescence signals (bottom) with corresponding brightfield data (top). (F) Positioning of combined image stacks from previous step (SIM/FM merge; bottom of column) in 2D X-ray mosaic (top). (G) 3D superposition of previous (bottom of column) with X-ray tomogram at ROI (top; lateral resolution of imaging beyond 60 nm; measured resolution at the B24 transmission X-ray microscope with the 40 nm objective). (H) The combined 3D stacks can now be further analyzed with image analyses packages such as Fiji (Schindelin et al., 2012) or segmentation algorithms such as SurVoS (Luengo et al., 2017) to extract statistical data on cellular structures or further combined with tomograms from adjacent ROIs to increase the 3D space imaged or segmented. (I) The resulting multi-channel stack has all imaging data correlated in 3D. A single slice from the middle of the tomograms is shown with the correlated 3D fluorescence volumes (in red for intracellular vesicles that contain molecules of interest and in green for reovirus; the scale bar is 1 μm). Superpositions in steps (D) and (E) were done with Chromagnon (Matsuda et al., 2018) and (F)–(H) with eC-CLEM (Paul-Gilloteaux et al., 2017); visualization and rendering were done with Fiji (Schindelin et al., 2012) and Chimera (Pettersen et al., 2004).

Cryopreservation lies at the heart of our workflow because it allows not only the immobilization and preservation of native cellular structures but also confers resistance to prolonged exposure to intense light during imaging. Cells to be imaged are typically grown as adherent monolayers, multilayers, or in suspension. Adherent cells are cultured on carbon films on gold EM grids (Agarwal et al., 1989) ideally with positional markers (finder grids). At this stage, fluorescent markers can be endogenously expressed or added to the culture media for uptake into the cells. Attachment, growth, confluency, and distribution of live cells in the media can be established via any number of conventional light and fluorescence microscopy methods prior to addition of fiducial markers (usually gold nanobeads), blotting (to remove excess media), and vitrification via plunge freezing in liquid nitrogen-cooled liquid ethane. Post-vitrification, grids are once again imaged in both bright field and fluorescence and mapped at the beamline on a cryostage-equipped light microscope with fluorescence detection capabilities, to examine cell morphology and to confirm absence of non-vitreous contaminants (Linkam Scientific CMS196M; Carl Zeiss AG Axio Imager 2; DIC 50×, 0.55 NA). At this point, the selected cells are ideally intact, with thin vitreous ice around them, and ROIs have been identified. Grids can now either be used as they are or clipped in autogrid holders (ThermoFisher) for greater stability. The cryoSIM is thereafter the first stop for high-resolution 3D imaging, followed by cryo-SXT on the same ROIs (Figure 4; STAR Methods). Because exposure to soft X-rays leads to atomic bond damage within fluorescence centers, X-ray imaging can never precede fluorescence imaging.

Samples are placed in standard Linkam cryoholders at the cryoSIM that is initially used to generate a brightfield transmission 2D mosaic of the grid holder structure, and ROIs are annotated on this mosaic as data collection takes places. 3D-SIM data are collected through the sample and along the z axis (4–12 μm depending on cell thickness). We have not observed any thawing of vitreous samples during data collection under the above-mentioned standard conditions and samples that have been used in cryoSIM imaging do not appear structurally different from other samples at the TXM. After SIM imaging, samples could also be used for cryo-electron tomography through a focused ion beam milling step (Medeiros et al., 2018), however, we have yet to fully explore this potential.

After cryoSIM data collection, grids are recovered from the Linkam holders and stored until they can be loaded into TXM holders (either conventional cryo-holders or autogrid TXM holders, the design for which was a kind gift by the Mistral beamline at the Spanish synchrotron, ALBA [https://intranet.cells.es/Beamlines/XM]). Once at the TXM, grids are examined and mapped with visible light to produce a new mosaic at the sample orientation (moving from one holder to the other results in random sample rotation for each microscope). The mosaics are then aligned with a semi-automated plugin at the beamline and the established ROIs from SIM data collection are recovered to allow X-ray data collection in the same regions. The samples are initially evaluated for susceptibility to radiation damage and uniformity of vitrification (semi-crystalline ice areas can be readily identified in X-ray projections). Next, a data collection strategy is devised on the basis of trial exposures in areas away from established ROIs in conjunction with the imaging requirements of each project. 2D X-ray mosaics are acquired at all ROIs covering single grid squares (imaging of 7 × 7 adjacent FOV at 16 × 16 μm will image a whole grid square in a standard EM grid) and these are used to decide on data collection areas. 3D X-ray data are collected as a tilt series and relayed in real-time to an automated pipeline by using IMOD Batchruntomo (Mastronarde and Held, 2017), and provided a sample has good contrast and fiducials (ideally >3 and well dispersed in the FOV), the data are reconstructed to tomograms by using IMOD’s weighted back projection, serial iterative reconstruction, and patch tracking options. The tomograms can then be used to further refine data collection strategy.

### *In silico* correlation of CryoSIM and CryoSXT data using eC-CLEM

**Figure 5:**
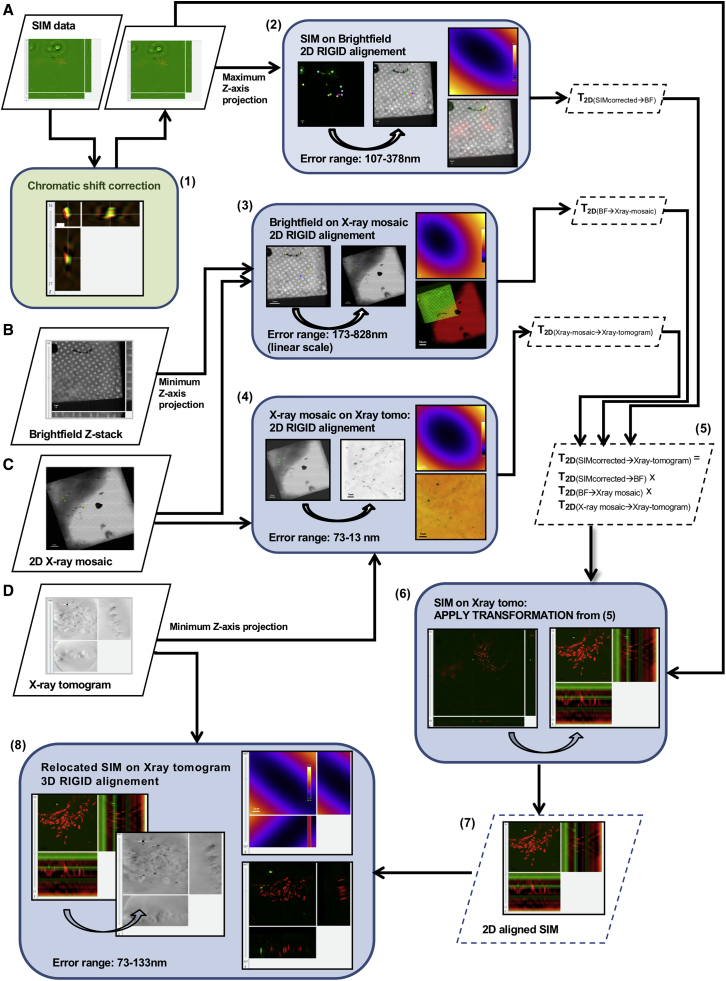
*In silico* correlative workflow for 3D volume alignment of CryoSIM data on CryoSXT volumes (A–D) The registration workflow consists of two steps: an initial 2D relocation of the SIM data on the X-ray tomogram and a final 3D alignment of volumes. Initially, the spatial relationship between (A) SIM datasets (corrected for chromatic 3D shift (1)) and (B) brightfield data are first computed by using a few features such as exemplified by the points of correlation (multi-colored dots) on the corresponding graphic (2). The other spatial relationships needed are between (B) bright field and (C) the large field of view 2D X-ray mosaic (3), and (C) the X-ray mosaic and (D) the X-ray tomogram (4). These 2D spatial relationships are then combined mathematically (2D transform matrix combination) to obtain the 2D spatial relationship between the chromatic shift-corrected 3D SIM and the high content X-ray tomogram (5). Given this 2D alignment (with estimated accuracy of 100–800 nm dependent on overall tomogram tilt), the 3D accurate registration is initialized (6), producing the rigid relationship between both corrected SIM volume and X-ray tomogram (7) that is used to fine align them in 3D (8). Each block of the workflow using eC-CLEM (Paul-Gilloteaux et al., 2017) is shaded blue and shows the source and target image used, with correlation paired-points used, and the associated error map (top left in each graphic) where color is linearly coding for the predicted average registration error (yellow is the maximum error and dark blue is the minimum in the range indicated in each bock) with the resulting 2D overlay (bottom left in each graphic). The graphic shaded in green denotes use of Chromagnon (Matsuda et al., 2018).

**Figure S3:**
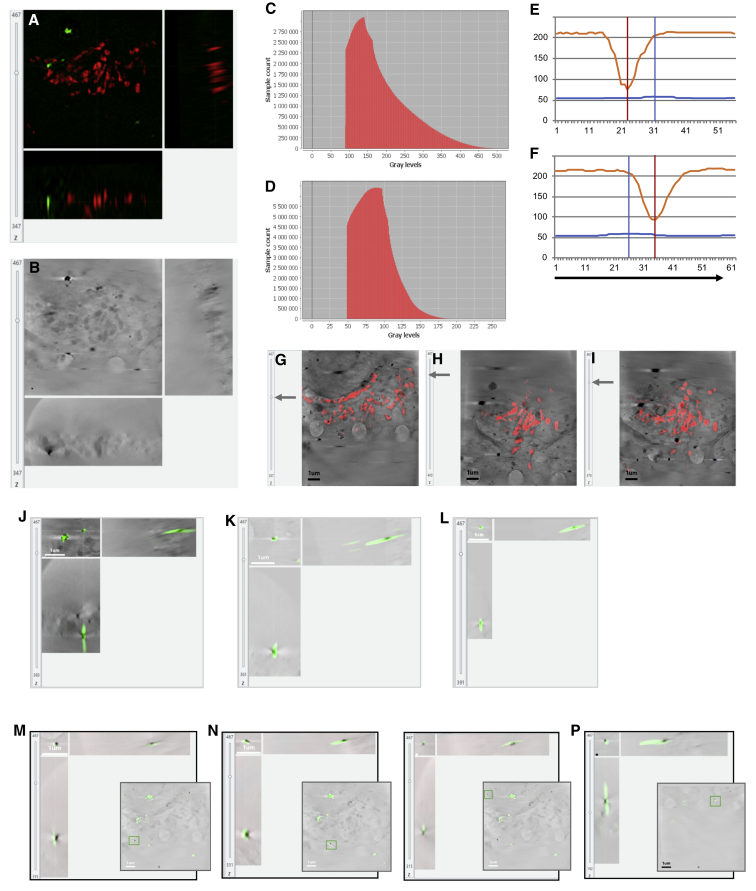
Accuracy of 3D correlation, related to Figures 4 and 5 In order to assess the accuracy of our 3D rigid registration procedure, a sample with two fluorescent features (gold beads and mitochondria) was used. It has been demonstrated that real alignment error can be predicted under the assumption of a correct model (rigid model used to match rigidly linked dataset) (Paul-Gilloteaux et al., 2017). We used this error prediction facility in eC-CLEM to assess correlated nanoparticle localization in both imaging modalities used (cryoSIM and cryoSXT). In (A) are orthogonal views of the 3D registered SIM dataset (green channel: Au 150 nm beads coated with Alexa488; red channel: mitochondria stained with Mitotracker red). The scale bars shown on XY views, apply in ZX and YZ views also (the generated data are isotropic with voxel size of 10x10x10 nm). Note that tomogram tilting shown in (B), was taken into account during 3D registration. In (B) are orthogonal views of the associated 3D X-ray tomogram used as target volume for 3D registration. The source volume was the pre-aligned 3D SIM dataset relocated in 2D as described in Figure 5. In (C) is the distribution of error when using only 5 beads (fewer points, localized within the SIM resolution) for the registration of the 9 beads visible in total within the fluorescence signal of this volume. This distribution is obtained by taking the histogram of the predicted average error of eC-CLEM error map which has been validated in Paul-Gilloteaux et al. (2017), but was checked against the beads here. X axis is the registration error, y axis is the number of voxels in this volume with this error. The error range (in 3D) was found to be between 80 and 500 nm, with 95% of the error below 350 nm, and 50% under 150 nm. In (D) is the distribution of error when using 15 unique points on mitochondria (more points, less accurately localized) for the registration. x axis is the registration error in nm, y axis is the number of voxel in this volume with this error. The error range (in 3D) was found to be between 50 and 300 nm, with 95% of the error below 130 nm, and 50% under 85 nm. In (E) and (F) are 2D line profiles along two beads (shown in J and K) in XY after overlay of 3D registered SIM and X-ray tomogram data. The orange line plot is the bead intensity profile in the X-ray volume and the blue line is the profile of the same bead in the 3D registered interpolated fluorescent data. The distance between the peaks of fluorescent signal and X-ray signal give an indication of the positional discrepancy in 2D of that bead. x axis is in pixels (1 pixel = 10 nm), y axis is the 8-bit converted intensity for X-ray and fluorescence signal. (G) (I) show 3 different slices of the 3D SIM-registered signal on their respective target X-ray positions. The superimposition of mitochondrial red signal on the visible X-ray mitochondrial trace show that the 3D rigid model used was correct, and the sample did not undergo any significant deformation. Scale bar is 1 μm. In (J–L) is a superimposition of the 3D-registered SIM green channel on the X-ray tomogram, cropped around beads used for the registration. Note that the beads have been tilted in the 3D SIM to match the tilt of the X-ray volume. Scale bar is 1 μm and is valid for XY and YZ representations. In (M–P) is the cropped area in orthoviews of the four beads not used for the registration. Each bead position is indicated in the bottom right 2D insert of the full FOV of the X-ray tomograph to highlight their relative distribution in the sample. As can be seen in particular for (N) and (P), the 3D discrepancy has to be considered to supplement 2D profile representations. Scale bar is 100 nm when not indicated.

Processed data from both high-end microscopes can be imported in eC-CLEM (Paul-Gilloteaux et al., 2017) initially for a preliminary *in silico* 2D relocation of all related datasets and thereafter for the refined 3D correlation of associated features (Figure 5). During this process, only rigid transformations are used to link the data (only rotation, translation, and isotropic scaling; the latter usually negligible). The main advantage of this approach is that imaging data are correlated in 3D without any local warping (Video S1). We have assessed the accuracy of our *in silico* correlative workflow by using test cryoSIM and cryoSXT data collected on U2OS cells that were stained with Mitotracker-Red and fiducialized by using 150 nm gold nanoparticles coated with green Alexa488 (Creative Diagnostics) (Figure S3). The accuracy of the first relocation step, using only 2D transformation, is dependent of the 3D tilting of the X-ray tomogram, because it uses projections for identifying beads location. In the worst case, accuracy in the 2D relocation was found to be in the range of 100–500 nm. This was sufficient in all cases to allow identification and correct 3D pairing of sample structures for the 3D fine registration step. The accuracy of the registration depends on the number of paired structures and of their localization accuracy (Paul-Gilloteaux et al., 2017). In order to assess the range of registration accuracy, two scenarios were tested: (1) using 5 nanoparticles (good localization accuracy, few paired structures), and (2) using 15 unique mitochondrial structures with unambiguous localization and distinct architecture. The registration accuracy was found to be between 50 and 130 nm when using 15 points, and between 80 and 350 nm when using 5 points for 95% of the 3D field of view. The accuracy was below 85 nm (5 nanoparticles) or 150 nm (15 mitochondrial structures) for 50% of the points used when located in the high accuracy central area of the data (error map included in Figure S3). These numbers were derived by using the statistical prediction functionality of eC-CLEM (error map) and were checked against nanoparticles not used for registration (4 beads visible in the FOV not used for the registration for validation purposes). The accuracy of the mitochondria 3D rigid matching (only 3D rotation and translation) shows no apparent deformation in the sample because of preparation vitrification or during imaging across modalities involved.

Video S1 3D Tomogram of the SIM and X-Ray Data Used to Assess Correlation Error Estimation, Related to Figure S3. Z stack motion through a U2OS cells with red fluorescent mitochondria and green fluorescent gold nanoparticles (diameter of 150 nm). Fluorescence and X-ray absorption signal were superimposed using rigid 3D alignment with eC-CLEM as described in the Results section.

### Proof of concept application of 3D cryo-correlation workflow: elucidating early events in reovirus infection

We have applied our imaging platform to better understand reovirus escape from endocytic vesicles. We have used the human bone osteosarcoma U2OS cell line expressing mCherry Galectin-3 (U2OS-Gal3; see STAR Methods for experimental details). Gal3 binds to extracellular carbohydrates and, within a normal cell, is distributed throughout the cytoplasm as it is produced and then trafficked to the cell surface. However, upon endosomal membrane disruption, extracellular carbohydrates that were internalized through endocytosis become accessible to Gal3 that can bind to carbohydrates within these vesicles. Hence, local concentrations of Gal3 can serve as useful indicators of endosomal membrane disruption (Maier et al., 2012).

**Figure 6:**
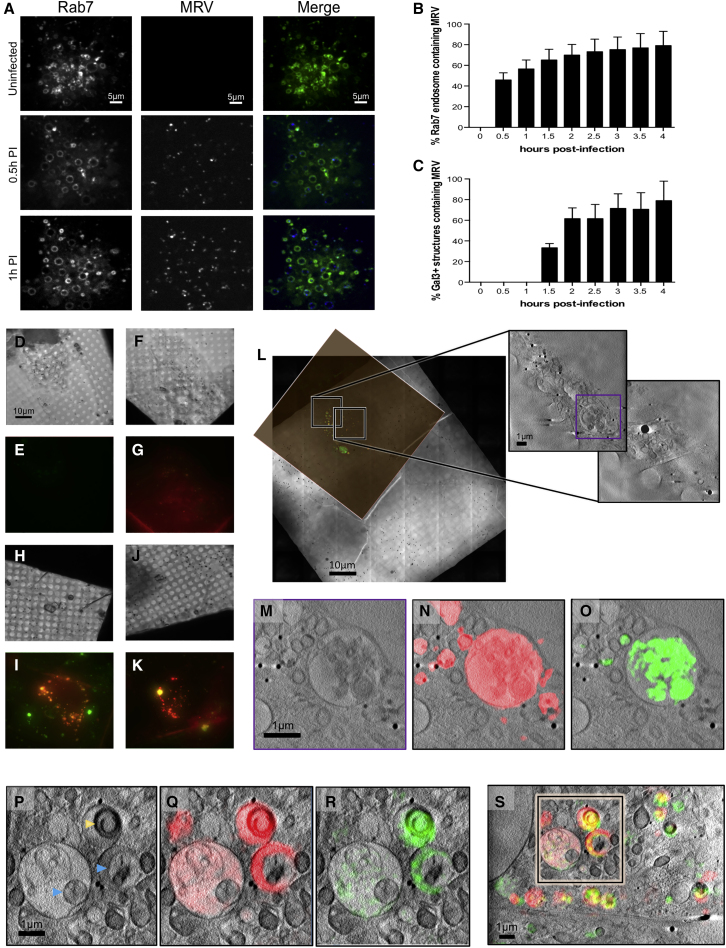
Tracking reovirus endosomal trafficking and escape (A) Confocal images of U2OS cells expressing late-endosome marker Rab7-eGFP infected with Alexa647-labeled reovirus at indicated times AI. (B and C) Mammalian reovirus (MRV) presence in late endosomes up to 4 h AI (B). Numbers of Gal3--positive endosomes containing MRV for the same period (C) (error bars indicate SD of n = 8 in B and n = 9 in C). (D–K) CryoSIM images of MRV labeled with Alexa488 in mCherry-Gal3 expressing cells showing the concentrations of Gal3 signal in distinct vesicles in red; brightfield and SIM slices respectively at: time 0 mock-uninfected (D and E); 1 h AI (F and G); 2 h AI (H and I); and 4 h AI (J and K). (L) Superposition of SIM processed data of a 4 h AI sample on the 2D X-ray mosaic of the same area and expansion of adjacent FOVs where two adjacent X-ray tomograms were collected and stitched. (M) Expanded region of interest (highlighted purple in L). (N and O) Same as (M) with Gal3 signal in red (N) and reovirus signal in green (O), respectively. (P–R) ROI from 2 h AI with either X-ray grayscale imaging alone or superposed wiith fuorescence signal: single z axis slice from the corresponding tomogram with Gal3 (red) and MRV (green) localization. Multi-vesicular bodies (MVBs) are clearly identifiable with distinct sub-compartments where Gal3 is concentrated (presumably the result of virus-carrying vesicles fusing with virus free late endosomes). Virus-induced carbon-rich substructures are marked by yellow arrows and vesicle areas within MVBs that remain impermeable to Gal3 marked by blue arrows. (S) Overview of the relevant 2 h AI region with both fluorescent signals overlaid.

**Figure S4:**
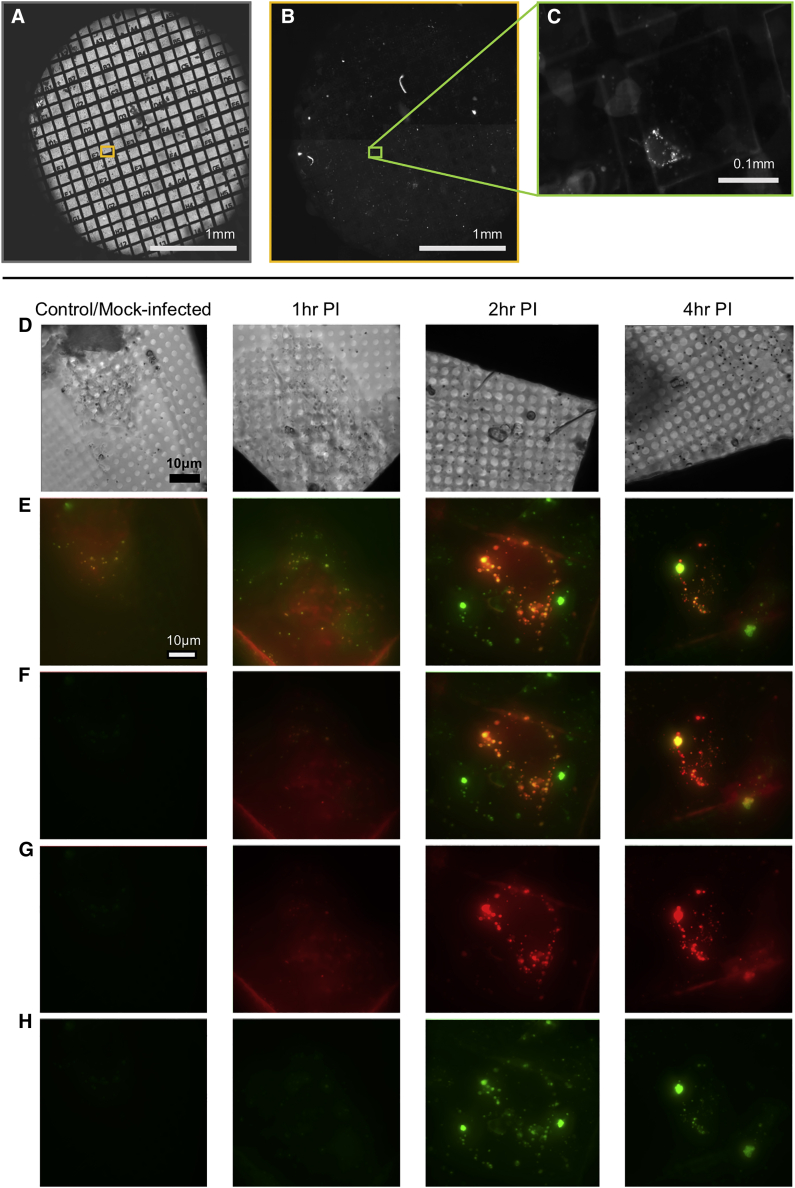
Visible light microscopy of the early stages of reovirus infection: conventional and super-resolution cryo-microscopy study, related to Figure 6 Conventional cryo-imaging using a AxioImager2 microscope coupled with a Linkam stage: In (A) is a brightfield mosaic of a 3 mm finder EM grid using white light; in (B) is the mCherry fluorescence in the same FOV and (C) shows an expanded view of an area in (B) (box outlined green) with evidence of subcellular structures with red fluorescence (mCherry-Gal3 in this case). Bright white spots are endosomes containing mCherry-Gal3 highlighting a cell in which tagged Gal3 is expressed. CryoSIM imaging of reovirus (Alexa488 in green) and Gal3 (mCherry in red) localization in cells at 0, 1, 2, and 4 h after infection: in (D) are brightfield images of representative cells (Z axis sum of minimum-intensity projections); in (E) is the maximum-intensity Z axis projection of reconstructed 3D SIM data (composite of both channels); (F) is the same as (E) but with fluorescence signal intensity normalized against the 4 h post-infection data to allow visualization of the relative increase in signal due to fluorophore localization; (G) shows the same view as above with mCherry (Gal3) fluorescence only in red; and (H) shows Alexa488nm (reovirus) fluorescence in green. Imaging in each column comes from the same sample/cell and all images are to the same scale.

We mapped the time course of reovirus localization in U2OS-Gal3 cells by transfecting them with Rab7-GFP (late endosomal marker) and subsequently infecting with fluorescently labeled T3D reovirus. Live-cell spinning-disc confocal microscopy was used to follow virus entry, the association of the fluorescent virus with the late Rab7-marked endosome, and the appearance of localized Gal3 upon endosomal rupture process. Our data show that within 1 h after infection (AI), more than 60% of virus particles are associated with Rab7-positive endosomes (Figures 6A and 6B). Importantly, by 2 h AI, 60% of Gal3-positive vesicles are associated with virus particles (Figure 6C). U2OS-Gal3 cells were then seeded on EM gold grids and infected with high titers of Alexa488-labeled T3D reovirus (see STAR Methods). Grids with infected cells were lightly blotted to remove excess media and plunged frozen into liquid nitrogen-cooled liquid ethane at different time points post-exposure to the virus (T = 0, 1, 2, and 4 h). All grids were first mapped through conventional cryo-microscopy in accordance with the workflow previously discussed (Figures S4A–S4C), and selected ROIs were 3D imaged first by using the cryoSIM (see STAR Methods). The resulting super-resolution imaging data extended the confocal study by capturing small Gal3-positive vesicles that are also positive for reovirus at 1 h AI (Figures 6D–6G), and these structures are seen to further increase in number, size, and signal intensity by 2 h AI, gradually forming large vesicle-like perinuclear structures (up to 3 μm diameter) by 4 h AI (Figures 6H–6K and S4D–S4H). It is therefore evident that, by 1 h AI, endocytosed reovirus particles have started disrupting endosomal membranes sufficiently to allow an influx of Gal3, and the affected endosomes continue to progress to the late stages of development.

**Figure S5:**
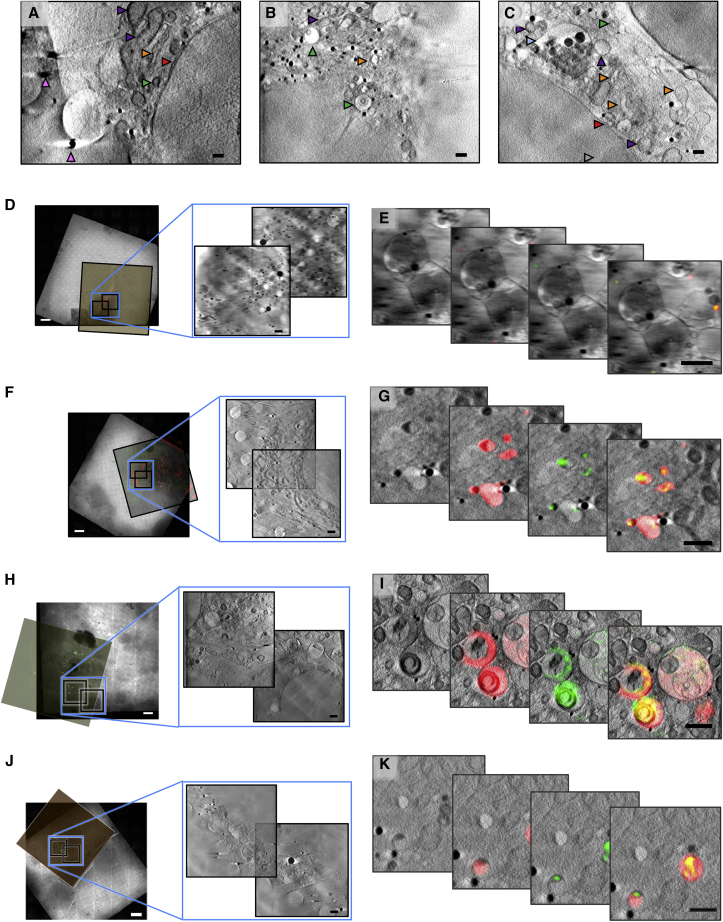
SXT and SIM imaging of mock-infected and reovirus-infected U2OS adherent cells, related to Figure 6 (A–C) Representative cross-sections from three cells, (A), (B), and (C), in the control population of U2OS cells. Cellular structures are identified with arrows: pink for the gold nanoparticle fiducials (250 nm), purple for mitochondria, red for the nuclear envelope, orange for the endoplasmic reticulum, green for MVBs, light blue for endo/lysosomes, gray for nucleoli. (D) 2D X-ray mosaics of a grid area with reovirus mock-infected U2OS cells with the corresponding SIM fluorescence slice aligned. The areas where higher resolution X-ray data were collected are outlined in blue (two ROIs ) and expanded. (E) Areas of focus in single slices from the 3D tomograms are shown as four overlapping frames with (from left to right): X-ray absorption only, composite of X-ray absorption and red fluorescence, composite of X-ray absorption and green fluorescence and composite of X-ray absorption with both fluorescence signals. (F and G), same as (D) and (E) respectively but for a cell population 1 h after infection. (H and I), same as (D) and (E) respectively but for a cell population 2 h after infection. (J and K), same as (D) and (E) respectively but for a cell population 4 h after infection. Black bars are 1 μm and white bars 10 μm. In (E), spurious signals represent background levels of fluorescence in U2OS cells; both green and red signals are perfectly co-localized, weak (relative to those observed later in the infection) and are not associated with any distinct cytoplasmic structures. In (G), 1h after infection, a number of small but distinct vesicles are both Gal3 and MRV positive while in (I), smaller endosomes have now merged to give rise to MVBs that have distinct compartments infused with Gal3 while others have not been similarly compromised (presumably arising from concatenation of membranes from MRV-free vesicles). Carbon-rich virus-induced domed structures are also seen in single vesicles. In (K), fluorescence is shown in smaller vesicles alongside large vesicle superstructures shown in Figure 6M.

**Figure S6:**
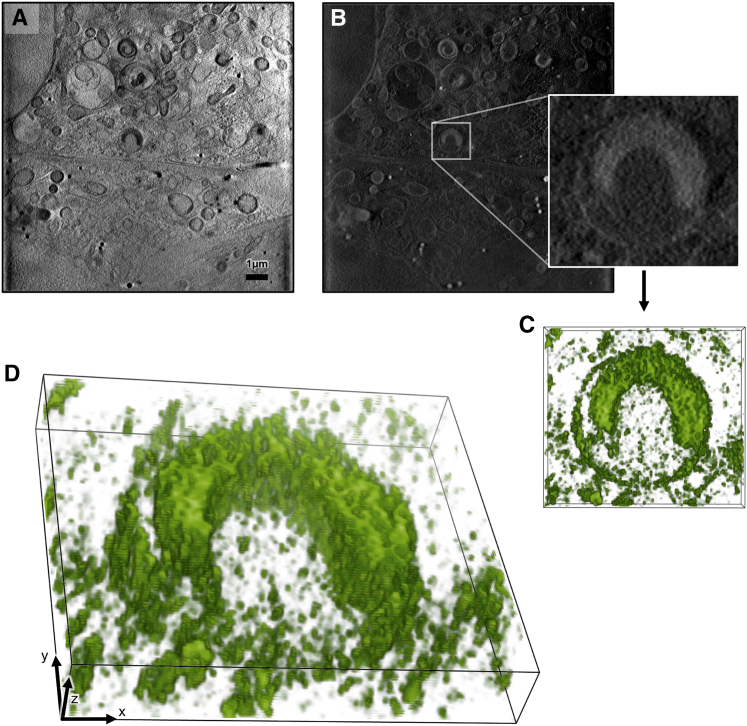
The 3D organization of virus-containing structures within cytoplasmic vesicles, related to Figure 6 (A and B) In (A), is a single Z axis slice of an ROI X-ray tomogram showing two infected cells at 2 h after infection (respective nuclei at top and bottom of left-hand side) and in (B) is the same with all pixel values inverted (necessary for volume rendering in Chimera) (Pettersen et al., 2004) with a sub-area expanded around a distinct semi-circular carbon-dense structure that is reovirus-positive. (C and D) In (C) is a surface rendering of a partial stack of the corresponding density looking down the Z axis and in (D), the same volume as (C), tilted to show the 3D structure. All images were generated with Chimera (Pettersen et al., 2004).

Having established the timeline of viral disruption of endosomes by using cryo-SIM (corroborated by our previous live cell confocal microscopy under similar conditions), we needed a high-resolution view of the landscape within infected cells that would unambiguously give us the 3D characteristics of the carrier organelles involved in virus trafficking, as well as their distribution and possible virus-induced damage. To achieve this goal, grids carrying vitrified infected cells, with ROIs already imaged at the cryoSIM, were transferred to the TXM where they were first mapped by using visible light via the accessory inline brightfield and fluorescence microscope before they were imaged via SXT (Figure 3; STAR Methods). For this project, we chose to image using the 40 nm objective of the microscope taking advantage of the extended FOV it affords us in order to collect large volumes of data and provide suprastructure context for the vesicles. The alternative of higher resolution imaging would have allowed us to potentially identify individual virus particles in the cytoplasm but over much reduced areas within cells (this high resolution aspect of the project is currently ongoing). SXT allowed us to unambiguously delineate vesicle structures across the cytoplasm including areas very close to the nucleus that would be largely inaccessible to other established nanometer-resolution techniques such as electron tomography (Figures 6L–6S and S5; Video S2). Using SXT, we observed: (1) populations of simple vesicles carrying viral load at all time points examined, and (2) some vesicles progressively growing larger in size and giving rise to multi-vesicular bodies (MVBs) localized away from the cell periphery and closer to the nucleus. MVBs were visible from 2 h AI (Figures 6P–6S) onward and continued to grow in size and complexity throughout the 4-h period after exposure to reovirus. We also observed (3) a subpopulation of vesicles that carried structured, carbon-rich features that form distinct domed structures (Figures 6P and S6). These structures were only observed in infected cells from 2 h AI and were not present in the cytoplasm of control cells (Figures S5A–S5C), suggesting that they are formed in response to viral presence. It is important to note here that all cells were exposed to high titers of reovirus that is reputed for its high infectivity, and although this is not necessarily dissimilar to the virus prevalence during an established infection physiologically (an infected cell can deliver hundreds of virions in its immediate environment), we cannot exclude the possibility that some of our observations might be the result of this “overloading.” Hence, the above mentioned domed structures could be the result of the accumulation of viral waste products because of the large numbers of particles intracellularly or it could represent a pathway for viral escape and/or release and can be the subject for further studies in the future.

Video S2 3D Tomogram of a Region of Interest in Cells 2 h After Infection Showing Part of the Cytoplasm of Two Infected Cells, Related to Figure 6. Respective nuclei are partially obvious at the top and bottom left hand corners; the cell-cell interface largely coincides with the tomogram x axis half way in the field of view. The movie starts at the top of the cells and traverses their volume until the carbon support film (characterised by the repeated 2/2 pattern of 2 μm diameter circles each 3 μm apart) and returns back through the cytoplasm to the top surface.

**Figure 7:**
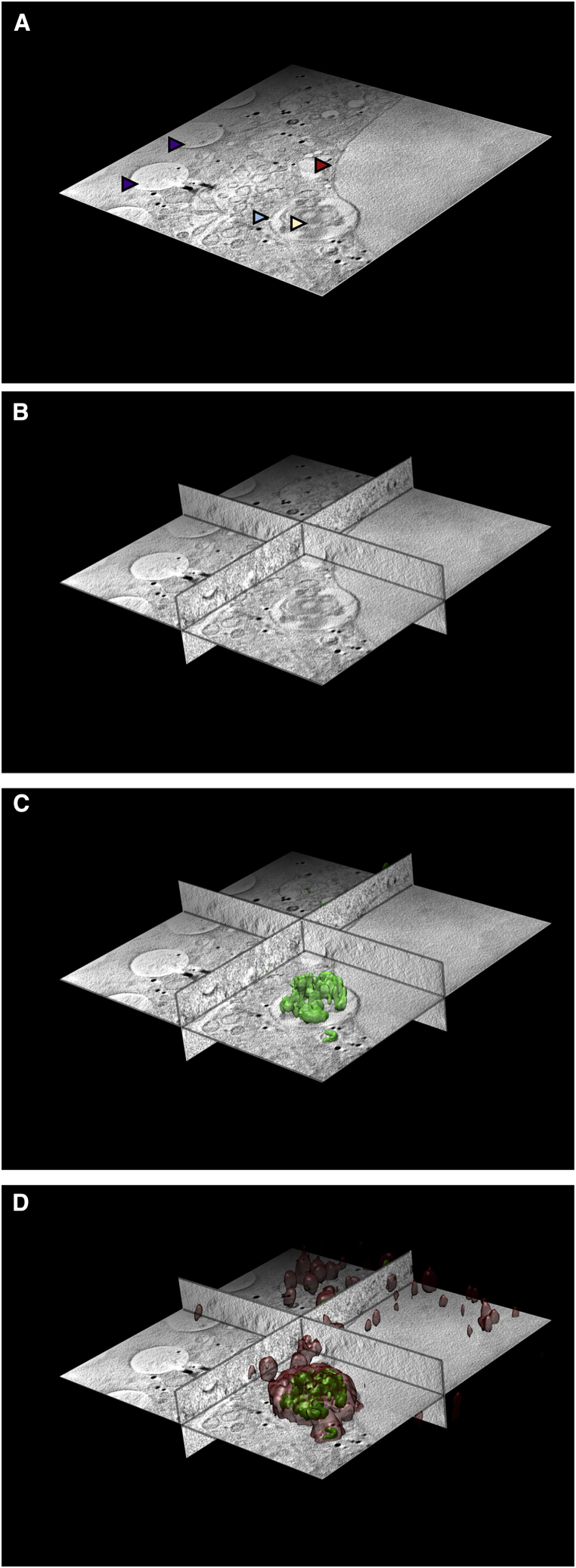
Correlation of X-ray and fluorescence data in three dimensions using the beamline B24 platform All the data shown here are from a U2OS cell vitrified 4 h AI with high titers of reovirus T3D. Viral components are fluorescently labeled with Alexa488 (green fluorescence), and there is also an endogenously expressed reporter molecule (Galectin-3) that is accumulated in vesicles carrying concentrated reovirus outer capsids and that carries the mCherry fluorophore (red fluorescence). (A) Z slice from the middle of a soft X-ray absorption tomogram (from beamline B24 using the 40 nm objective) of a U2OS cell with the nucleus on the right side of the figure (red arrow; the two leaves of the nuclear membrane are seen clearly) and the cytoplasmic area with a substantial multi-vesicular body/endolysosome (pale blue arrow), containing folded carbon-dense material given the increased X-ray absorption in that structure (pale yellow arrow). In the thinner areas of the distal cytoplasmic region, several holes can be seen as part of the support film used for cell attachment (deep purple arrows). (B) Ortho-slices of (A), showing a representative portion of the volumetric data. (C) 3D rendering of the cryoSIM recorded structures displaying green fluorescence within and around the endolysosome. (D) 3D semi-transparent rendering of all cytoplasmic vesicles that contain red Galectin-3 fluorescence (data also collected on the cryoSIM) and are seen as an indication of viral presence in those vesicles. All images were generated with Chimera (Pettersen et al., 2004).

**Figure S7:**
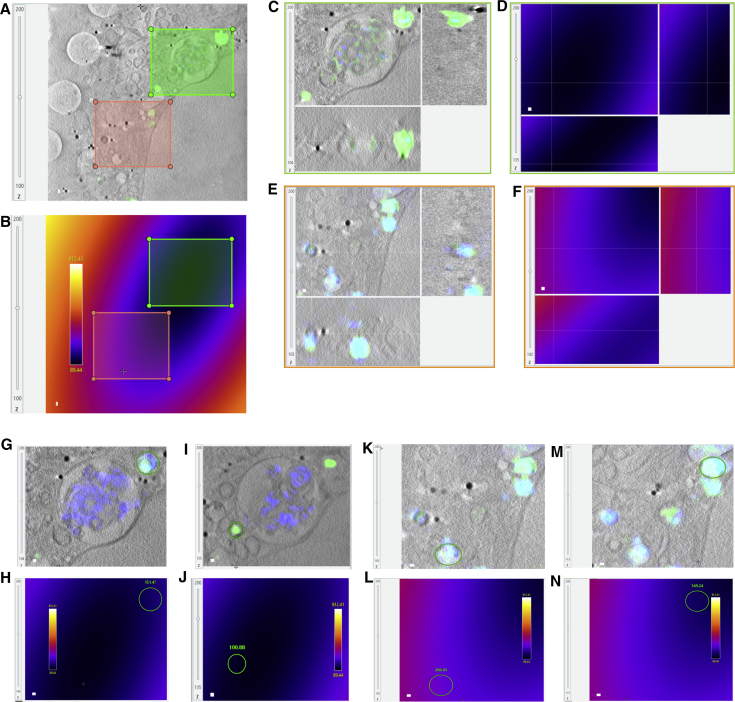
Estimation of error on reovirus datasets, related to Figures 6, S3, and S5 (A and B) In (A) is an XY slice of the 3D X-ray tomogram (extracted from 4 h post-infection datasets) and in (B) is the corresponding slice of the 3D error map generated using the point-set used for registration. The color codes for predicted average error is in nm, ranging from 89 to 813 nm in the full volume (two sub regions are selected for best and possible worst accuracy in this view (green shaded and red shaded respectively). Best accuracy areas overall lie near the gravity center of the point-set used for registration, dark blue). The scale bar is 100 nm. (C and D) In (C) are orthoviews of a crop of the merged dataset in the green sub region and in (D) is the corresponding crop in the error map. This area given the registration used, corresponds to the best accuracy we can obtain in this particular volume (89 to 150 nm). Scale bar is 100 nm. (E and F) In (E) is an orthoview of a crop of the merged dataset in the red sub region of (A), and in (F) is the corresponding crop in the error map. This orthoview is centered in an area with a predicted accuracy of 300 nm, which can be seen in the discrepancy in Z on the focused endosome. Scale bar is 100 nm. (G–N) Example of accuracy measured on finer areas within the error map for different endosomal arrangements. The circled areas indicate local 2D areas selected for which average predicted errors was measured, and their matching position on the merged dataset. The predicted error is displayed above these in the error map in nm. (G)–(J) and (K)–(N) are taken the green and red sub regions respectively. The scale bar is 100 nm.

Once all data were collected, we used eC-CLEM (Paul-Gilloteaux et al., 2017) to align in 3D all associated imaging that allowed us to further expand the information content available (Figures 6M–6S and 7). The accuracy of the correlation was dependent of the number of identified structures in common in both X-ray and fluorescence 3D data. Assessment of the accuracy of our *in silico* correlative work demonstrated that the error range in ROIs was <100 nm (10 voxels in the X-ray tomograms), and areas of lower accuracy could be reliably predicted (Figure S7).

## Discussion

To address the current limitations of imaging relatively thick cryo-preserved biological samples at near-physiological states to tens of nanometers resolution, we have developed a correlative imaging platform for data collection on cryo-preserved vitrified samples (primarily cell populations, adherent, or in suspension). In summary, the workflow described here focuses on cryo-preserved cells mounted on flat EM grids. The latter are at first mapped by using conventional brightfield cryo-fluorescence imaging where the areas of interest are identified before 3D imaging data are collected, first by using our cryo-3D-SIM system and then at a soft X-ray full-field transmission microscope. Data are then processed and correlated to produce 3D imaging volumes that contain both chemical localization and ultrastructural organization information enabling the meaningful and unambiguous interpretation of biological features. Uniquely, our platform has been developed to incorporate two cutting-edge imaging methods with the aim of delivering correlative imaging of unperturbed biological samples that is accessible, fast, and reliable. It utilizes established sample preparation protocols and transitions between imaging modalities without alterations in native sample physiology and characteristics. The microscopes used are both free at the point of access for academics, and in the case of the cryoSIM, designed for ease of construction at any other facility using “off the shelf” components. Comprehensive imaging with both microscopes can be performed in a matter of days and large swathes of cellular landscapes can be captured rapidly with processing largely automated.

The methods implemented here have been carefully selected to address requirements for both high resolution cellular imaging and related chemical localization. Cryo-SXT is currently the only method that allows the visualization of cellular substructure through thick areas, in fully hydrated cells without the need of fixation, staining, or sectioning and their potential associated artifacts in membrane structures such as endocytic vesicles. To complement the information content of our X-ray tomograms, we selected 3D-SIM as the super-resolution imaging technique of choice, because it has distinct advantages for cryo-imaging of biological samples suitable for cryo-SXT. It requires relatively low illumination levels while still giving up to twice the resolution of conventional widefield or confocal fluorescence imaging. It is also capable of imaging 3D samples over a relatively large depth and has the lowest total light dose among the super-resolution techniques. SIM imaging can be performed straightforwardly by using standard dyes, or fluorescent fusion proteins, and it is simple to image a single sample by using multiple fluorescent channels. The fundamental tenets that drove development of our workflow were that samples were at no point altered during imaging or across microscopies, and the resulting modalities would be technologically transparent, easily accessible, and where possible, easily reproducible.

We applied our platform toward the study of reovirus vesicle egress. Starting from confocal analyses we showed that by 1 h AI the virus is contained within a distinct endosomal population whereas by 2 h AI, 60% of these had been permeated by Gal3 that led to the identification of candidate time points for core particle release. Infected cell populations were then captured through plunge-freezing at 2–4 h AI and imaged using the cryoSIM that provided us with high-resolution 3D imaging on samples that could be directly transferred to SXT for the unambiguous high-resolution mapping of the vesicles involved in reovirus shuttling. Using 3D cryo-SIM, endosomes (within infected cells) carrying virus were easily identified. We were able to push the timeline of membrane disruption and subsequent Gal3 influx to 1 h AI and confirm that affected endosomes present as spherical vesicles that enlarge and develop into MVBs while progressing to the perinuclear area within the first 3 h AI. Therefore, we now have a clear timeline for viral escape and the apparent transport of viral material through the conventional endosomal pathway.

SXT of the same vesicles gave definitive 3D views of their architecture and their distribution in the cytoplasm of infected cells at the selected time points and indicate that initial endocytosis is followed by smaller vesicles merging to form progressively larger vesicles while their outer membrane carbon absorption remains relatively uniform at different time points.

Correlated fluorescence and X-ray data confirmed the presence of localized Gal3 within virus-bearing vesicles suggesting that their membranes have been modified enough (presumably through pore formation) to allow influx of cytoplasmic proteins and possibly other small cellular constituents from 1 h AI. Crucially, the implication of this observation is that core particles are able to escape into the cytoplasm at this time point AI. Virus egress does not disrupt the overall ultrastructure of virus-laden vesicles because none of the Gal3 and reovirus-positive vesicles appears misshapen or deflated and have no ruptures of their membranes that are contiguous across the circumference of each vesicle. Assuming that virus-induced pores shuttle viral cores (a sizeable 60 nm or more) across the endosomal lipid bilayer, the observation that the integrity of the affected endosomes is not structurally compromised is suggestive of selective shuttling of core particles with pores closing or reducing in size, when not in use, to ensure endosomes remain apparently undamaged. This would further aid in shielding the virus from detection during the early stages of infection. By 2 h AI, virus-positive endosomes are becoming larger, more complex, and multi-vesicular in structure and are found closer to the nucleus, indicating that they are trafficked for either recycling within or expulsion from the host cell. It is striking that, using our correlative approach, distinct domed structures observed with SXT within single vesicles in the cytoplasm by 2 h AI (Figures 6P and S6) can now be unambiguously identified, via the cryo-SIM data correlation, as virus associated, suggesting that they are vesicle-sequestered structures likely enriched with the remnants of reovirus outer capsids. Future work will concentrate on further analysis of biomarkers on the endosomes under study, tracking of lysosome merging and the full characterization of the enveloped domed structures observed at 2 h AI as well as their origin and possible role in infection or pathogen clearance.

It is important to note that our reovirus-related correlative data are rich in content (Videos S3 and S4) and could not have readily been acquired by other microscopy methods without sectioning or other potentially artifact-inducing sample processing. Although each of the imaging modalities used contributes independently to our understanding of the system under study, once combined within the unique correlation scheme presented here, they allow us to characterize and further understand medically relevant biological process such as the early events of viral infections. This study demonstrates the power of correlative imaging approaches in revealing structural and dynamic properties of complex cellular processes down to a resolution of tens of nanometers. Cryo 3D SIM-SXT correlation brings together two powerful volume imaging techniques that will become invaluable for investigations in a wide range of biological samples from cell cultures to tissues. The method could also function alongside other imaging techniques such as electron microscopy and provide an exemplar for further opportunities to integrate and develop innovative multi-modal techniques and tools for cellular high-resolution imaging in the future.

Video S3 Correlative 3D Data of X-Ray Absorption and Fluorescence Imaging, Related to Figure 6. As Video S1 but including fully correlated 3D green fluorescence (reovirus) and 3D red fluorescence (Gal3) identifying unambiguously which of the organelles visible in the X-ray tomogram are carrying reovirus and whether or not they contain highly concentrated Gal3. Note that although both cells are infected given the prevalence of green fluorescence the top cell does not express Gal3 and therefore shows no red fluorescence.

Video S4 Composite 3D Volume of a Region of Interest in an Infected Cell 4 h After Infection Showing the Virus Concentrated in a Large Late Endosome/Endolysosome Type Vesicle, Related to Figures 6 and 7. The nucleus of the cell is partially obvious at the bottom right corner of the field of view with a large vesicle nestling against the nuclear envelope. The latter contains carbon-dense structures that appear to be folded several times and display concentrated viral signal. The movie starts at the carbon support layer and traverses the cytoplasm toward the top of the cell.

## STAR★Methods

### Key Resources Table

**Table:** 

REAGENT or RESOURCE	SOURCE	IDENTIFIER
**Antibodies**		

Alexa488 NHS Ester	Thermo Fisher Scientific	Cat#A20000
Alexa647 NHS Ester	Thermo Fisher Scientific	Cat#A20106

**Bacterial and Virus Strains**		

Reovirus type 3 (strain Dearing) (T3D)	Ramig et al., 1977	https://doi.org/10.1128/JVI.22.3.726-733.1977

**Chemicals, Peptides, and Recombinant Proteins**		

Trichlorofluoromethane	Sigma-Aldrich	Cat#48541
Cesium chloride	Sigma-Aldrich	Cat#C4036
Dulbeccos’ modified Eagle medium (DMEM)	Thermo Fischer Scientific	Cat#41966029
Fetal Bovine Serum (FBS)	Capricorn	Cat#FBS-11A
Penicillin/streptomycin	Thermo Fischer Scientific	Cat#15070063
Joklik MEM medium	Sigma-Aldrich	Cat#M0518
L-Glutamine	Thermo Fischer Scientific	Cat#25030081
Neonatal calf serum	Thermo Fischer Scientific	Cat#26010-74
Mitotracker Red FM	Thermo Fischer Scientific	Cat#M22425

**Critical Commercial Assays**		

Zeba Spin Desalting Columns, 7K MWCO	Thermo Fischer Scientific	Cat#89882

**Deposited Data**		

U2OS- reovirus mock Infected (Area 1)	EMPIAR: EMPIAR-10412	BioImage Archive: S-BIAD17
U2OS- reovirus mock Infected (Area 2)	EMPIAR: EMPIAR-10413	BioImage Archive: S-BIAD17
U2OS- reovirus 1hafter infection (Area 1)	EMPIAR: EMPIAR-10414	BioImage Archive: S-BIAD18
U2OS- reovirus 1hafter infection (Area 2)	EMPIAR: EMPIAR-10415	BioImage Archive: S-BIAD18
U2OS- reovirus 2hafter infection (Area 1)	EMPIAR: EMPIAR-10416	BioImage Archive: S-BIAD19
U2OS- reovirus 2hafter infection (Area 2)	EMPIAR: EMPIAR-10417	BioImage Archive: S-BIAD19
U2OS- reovirus 4hafter infection (Area 1)	EMPIAR: EMPIAR-10418	BioImage Archive: S-BIAD20
U2OS- reovirus 4hafter infection (Area 2)	EMPIAR: EMPIAR-10419	BioImage Archive: S-BIAD20

**Experimental Models: Cell Lines**		

L-cells	ATCC	Cat#CRL-6364
U2OS cells	ATCC	ATCC HTB-96
U2OS cells expressing galectin3-mCherry	Maier et al., 2012	https://doi.org/10.1128/JVI.01428-12
BSC-1 cells	ATCC	ATCC CCL-26

**Recombinant DNA**		

eGFP-Rab7 plasmid	Choudhury et al., 2002	Addgene; Cat#12605

**Software and Algorithms**		

SoftWoRX 6.5.2	GE Healthcare	N/A
Chromagnon	Matsuda et al., 2018	https://github.com/macronucleus/chromagnon
Fiji	Schindelin et al., 2012	https://imagej.net/Fiji
SIMcheck	Ball et al., 2015	https://github.com/MicronOxford/SIMcheck
IMOD package (version 4.9.2)	Kremer et al., 1996	https://bio3d.colorado.edu/imod/
eC-CLEM	Paul-Gilloteaux et al., 2017	http://icy.bioimageanalysis.org/plugin/ec-CLEM
Chimera	Pettersen et al., 2004	http://www.cgl.ucsf.edu/chimera/
SurVoS	Luengo et al., 2017	https://diamondlightsource.github.io/SuRVoS/
Linkam’s LINK software	Linkam Scientific	https://www.linkam.co.uk/link-controlsoftware
Cockpit	Micron, Oxford University	https://github.com/MicronOxford/cockpit
Python Microscope	Micron, Oxford University	https://www.python-microscope.org/

**Other**		

TEM grids	Quantifoil	Cat#AU G200F1 finder
Grid holders	Thermo Fisher Scientific	Model#AutoGrid
250 nm gold nanoparticle fiducials	BBI Solutions	Cat#SKU EM.GC250
150 nm gold nanoparticles coated with red Alexa 488	Creative Diagnostics	Cat#GFL-150
PS-Speck Microscope Point Source Kit	Thermo Fisher Scientific	Cat#P7220
CryoSIM 405 nm laser	Omicron-Laserage	Cat#Bluephoton® TA Cat#Deepstar® Series – 375 nm – 488 nm
CryoSIM 488 nm laser	Omicron-Laserage	Cat#Bluephoton® TA Deepstar® Series – 375 nm – 488 nm
CryoSIM 647 nm laser	Omicron-Laserage	Cat#Redphoton® TA Deepstar® Series – 635 nm – 1060 nm
CryoSIM 561 nm laser	Cobolt	Model#Sapphire laser
CryoSIM 520/35 nm filter	Semrock	Cat#BrightLine® single-band bandpass filter, FF01-520/35-25
CryoSIM nematic liquid crystal	Meadowlark Optics	Cat#PDM512
CryoSIM polarization rotator	Meadowlark Optics	Cat#LPR-100-λ
CryoSIM multi band dichroic	Chroma	Cat#ZT405-488-561-647-22.5deg (this paper)
CryoSIM 100X air objective	Nikon	Model#CFI TU Plan Apo EPI 100X, 0.9 NA
CryoSIM cameras	Oxford Instruments	Cat#Andor iXon Ultra 897
CryoSIM & Axioimager Linkam cryostages	Linkam Scientific	Cat#CMS196M LED Cryo Correlative Stage
Axioimager microscope	Carl Zeiss AG	Model#Axio Imager 2
Axioimager 50x objective	Carl Zeiss AG	Model#50x / 0,55 DIC
Confocal microscope	PerkinElmer	N/A
Confocal 60x objective	Nikon	Model#1.49 NA, Apo TIRF
Confocal 100x objective	Nikon	Model#1.4 NA, Plan Apo VC
Confocal camera	Hamamatsu	Cat#CMOS, Orca Flash 4
TXRM microscope	Carl Zeiss X-ray Microscopy, Inc.	Cat#UltraXRM-S220C
TXRM photon detector	Princeton Instruments	Cat#Pixis1024B CCD
TXRM camera	Teledyne	Cat#Retiga 4000R

### Resource Availability

#### Lead Contact

Further information and requests for resources and reagents should be directed to and will be fulfilled by the Lead Contact, Maria Harkiolaki (maria.harkiolaki@diamond.ac.uk).

#### Materials Availability

This study did not generate new unique reagents

#### Data and Code Availability

Original imaging data referenced in the manuscript is deposited at the BioImage Archive (https://www.ebi.ac.uk/biostudies/BioImages) and EMPIAR (https://www.ebi.ac.uk/pdbe/emdb/empiar/) is. The accession numbers for the data are EMPIAR: EMPIAR-10412, EMPIAR-10413, EMPIAR-10414, EMPIAR-10415, EMPIAR-10416, EMPIAR-10417, EMPIAR-10418, EMPIAR-10419 and BioImage Archive: S-BIAD17, S-BIAD18, S-BIAD19 and S-BIAD20.

### Experimental Model and Subject Details

#### Cell lines and culture conditions

##### Cell culture and cell lines

U2OS cells (ATCC), or U2OS cells expressing galectin3-mCherry (a kind gift from Harold Wodrich, Bordeaux) were kept in Dulbeccos’ modified Eagle medium (DMEM) (Thermo Fischer Scientific) containing 10% Fetal Bovine Serum (FBS) (Capricorn) and 1% vol/vol of penicillin/streptomycin (Thermo Fischer Scientific) at 37 °C and 5% CO_2_. Within the U2OS population presented in this work, only a proportion of the cells expressed endogenously fluorescent Gal3 (designed to be selected under antibiotic control; no antibiotics were used in this case leading to a mixed population). Suspensions of L-cells (ATCC) for virus production were maintained in Joklik MEM medium (Sigma-Aldrich) supplemented with 1% L-Glutamine (Thermo Fischer Scientific), 2% FBS, 2% Neonatal calf serum (Thermo Fischer Scientific) and 1% penicillin/streptomycin at 35°C. BSC-1 cells (ATCC) were cultured and maintained in DMEM supplemented with 10% FBS and 1% penicillin/streptomycin.

### Method Details

#### CryoSIM optical setup

Excitation path: The illumination path consists of dichroics and mirrors to combine 4 linearly vertically polarized illumination beams (405 nm, 488 nm, and 647 nm Omicron Deepstar lasers; 561 nm Cobolt Sapphire laser; the polarization angles are carefully matched using half-wave plates). The combined light is then passed into a telescope section with a pinhole at the focus to act as a spatial filter. This infinity focused beam is delivered to the main beam height (200 mm) and passed through a square aperture onto a variable phase delay nematic liquid crystal on silicon SLM (Meadowlark Optics, 512×512 SLM, PDM512) used as a phase grating to produce structured illumination patterns. The light reflected from the SLM is refocused by a lens (all lenses are achromatic doublets) to produce an array of diffraction spots. An aperture at the focus of this lens blocks light from higher order diffraction spots, allowing orders 0 and ± 1 through. These pass through a half waveplate and then an LCD based polarization rotator (Meadowlark Optics, LPR-100λ) to maintain radially linearly polarized spots (necessary to ensure good structured illumination pattern contrast in the image plane). Another telescope transfers the spot images on to a silver coated mirror, passing through a primary dichroic (Chroma ZT 405-488-561-647-22.5deg). This dichroic transmits the excitation light and reflects the emitted fluorescence. The reflected beam passes through another telescope and reimages the focused diffraction spots in the back focal plane of the objective. A 45° mirror reflects the beam down onto the sample through an 100X air objective (Nikon, CFI TU Plan Apo EPI 100X, 0.9 NA, 2 mm working distance).

Emission path: Fluorescence signal from the sample is collected by the objective and transmitted back from the 45° mirror, through a 1:1 telescope and reflected by the silver-coated mirror upstream then separated from the excitation light by the primary dichroic. The emitted light is then reflected toward the cameras (Oxford Instuments, Andor iXon Ultra 897) by a broadband dielectric mirror and a final telescope which magnifies the image to optimize the camera pixel size to the optical resolution. There is a secondary dichroic, that splits the light before the detectors. Each detector has a dedicated filter wheel to allow selection of different emission channels. All hardware is controlled by two open source software packages, Cockpit and Python Microscope. These packages allow control of complex microscope systems in real time and provide a simple, user-friendly interface.

SIM resolution doubling is achieved by the structured illumination encoding the whole image content around each of the 5 spots, in the Fourier representation of the image, induced by the structured illumination. This information is separated and the copies of the image information are then all moved to the origin. If the highest frequency stripes are right at the edge of the observable region in the microscope, shifting these data to the origin moves the corresponding data on the opposite side of the Fourier transform to double its original frequency, hence doubling the highest detectable spatial frequency, and hence the resolution. Using lower frequencies stripes will mean these data are moved less far in Fourier space and produces a reduced resolution increase. In order to ensure adequate signal to noise in the high frequency information, we set the stripe width to larger than the finest possible, as this significantly increases the amplitude of the high frequency information returned (Figure S1). As a compromise between the achievable resolution increase in SIM and the reduction of signal with progressively finer stripe widths, we chose a stripe width of 396 nm with 488 nm excitation. This relatively coarse stripe width moves the information from the Moiré fringes in the SIM Fourier images away from the edge of the observable region, toward the center. Given that during reconstruction, the information is moved less far in Fourier space, a smaller resolution increase can be achieved. The 396 nm stripes we use with 488 nm excitation produce a maximal possible resolution in the SIM reconstructions of 190 nm, as opposed to the theoretical maximum of 180 nm with 525 nm emission light. However, with this small resolution reduction we roughly double the intensity of this shifted information content dramatically increasing the signal to noise ratio in the highest resolution information in the reconstructions. With a theoretical resolution of 190 nm, we reliably produce real images with resolutions of 200 nm. A full list of theoretical and achieved resolutions is given in Table S1.

CryoSIM layout, optics, hardware and software can be found at Dobbie et al. (2020) DOII).

#### SXT setup

The TXM is illuminated by synchrotron radiation supplied by a bending magnet, focused by a toroidal mirror and conditioned with a plane grating monochromator and exit slit module that can deliver highly monochromatic beam at 500 eV (Figures 2A–2C). This beam forms a secondary light source which is delivered to the microscope and focused by a glass capillary condenser lens onto the sample. A zone plate objective focuses the resulting projections onto a highly sensitive photon detector (Princeton Instruments, Pixis1024B CCD). Samples on standard cryo-EM grids are placed into the X-ray microscope using a transfer chamber that facilitates transition from liquid nitrogen storage under atmospheric pressure to active cooling via conduction in high vacuum (10^−6^ to 10^−8^ mbar, essential as soft X-rays have poor penetration at atmospheric pressure). Because of the divergence of synchrotron bending magnet sources, the size of the focused X-ray beam at the sample position is approximately 1.2 μm. To illuminate the maximum field of view (FOV) possible, the condenser oscillates following a Lissajous pattern, matching the acceptance of the 40 nm zone plate objective while illuminating an augmented 16x16 μm^2^ area at the sample plane (10x10 μm^2^ for the 25 nm objective) (Figure 2D). Samples at the imaging position are close to both the capillary condenser and the zone plate objective, circa 6 mm and 5 mm from each (Figure 2E), limiting specimen tilt to a maximum of ±70°, which leads to missing wedge artifacts. This may be mitigated in part by tilting around two orthogonal axes (Mastronarde, 1997) and SXT data collection at beamline B24 can be done using one or two axes depending on project and sample requirements. The zone plate objectives of the system are designed to focus soft X-rays; they are made of a series of concentric metal rings of radially decreasing width that are installed approximately 5 mm after the sample (beam divergence of 1.7 mrad; zone plate diameter of 150 μm). The resolution,δ, of this microscope depends on its optics and specifically the width of the outer most zone of its objective ($\delta =1.22\;\Delta r_{n}$ for incoherent imaging).

#### Virus production and purification

Reovirus T3D strain was produced by infecting suspension of L-cells with a T3D stock originally obtained from B. N. Fields. Virus particles were pre-purified from L-929 cells (ATCC) by sonication and freon (1,1,2-trichloro-1,2,2-trifluoroethane; Sigma-Aldrich) extraction; virus particles were then purified through ultracentrifugation on Cesium Chloride (CsCl) (Sigma-Aldrich) gradient and stored in virus buffer (150 mM NaCl, 10 mM MgCl2, and 10 mM Tris-HCl, ph7.5) as previously described (Fratini et al., 2018).

#### Virus labeling

100 μL of reovirus particles (from 10^13^ particles/ml stock) were mixed with 0.4 μL of Alexa488 or Alexa647 NHS Ester (Thermo Fisher Scientific) or Alexa647 NHS Ester (8mM starting concentration) for 1h at room temperature (RT). To remove unbound fluorophores, virus particles were then purified by gel filtration (Zeba Spin Desalting Columns, 7K MWCO, Thermo Fischer Scientific).

#### Live-cell imaging of virus infection

U2OS wild-type or stably expressing mCherry-Gal3 were transfected with an eGFP-Rab7 (Addgene) expressing plasmid 16 h prior to imaging. MRV labeled with Alexa647 was added to cells and then imaging was started. Live-cell imaging was performed with an inverted spinning-disk confocal microscope (PerkinElmer) using oil immersion objectives (60x, 1.49 NA, Apo TIRF, Nikon or 100x, 1.4 NA, Plan Apo VC, Nikon) and a CMOS camera (Orca Flash 4, Hamamatsu). Cells, objectives and microscope stage were kept at 37°C and 5% CO_2_ through the presence of an environment-control chamber. Cells were imaged in 0.5 μM stacks 5min apart for 180 min.

#### Virus infection for X-ray imaging

U2OS cells stably expressing mCherry-Gal3 were seeded onto TEM grids (Quantifoil AU G200F1 finder) 16 h prior to infection. MRV labeled with Alexa488 was added to cells along with 250 nm gold nanoparticle fiducials (BBI Solutions) and grids were frozen in liquid nitrogen-cooled liquid ethane using Leica EM GP2 plunge freezer with a 2 s blotting time at 1h intervals. BSC-1 cells were also seeded on TEM grids 16 h prior to infection. Cells were infected with MRV at an MOI of 100 and 16 h after infection grids were frozen in liquid nitrogen-cooled liquid ethane using a Leica EM GP2 plunge freezer with a 2 s blotting time.

#### Monitoring infection status

Infection prevalence was confirmed via confocal microscopy (presence of green fluorescence virus components intracellularly) before sample vitrification but also with inspection of the same signal once vitrified using a Linkam cryo-stage on a conventional microscope (AxioImager2) using a 50x objective (0,55 DIC). The latter allowed us to map grids (using the Linkam’s LINK software) and assess their quality with respect to population density, vitrification, presence of fluorophores and grid integrity.

#### Cryo-SIM imaging and high-resolution data reconstruction

Vitrified samples on grids were transferred to the cryoSIM and brightfield imaging was first employed to generate mosaics, where individual cells were evaluated based on cell location (likely to allow data collection on both this instrument and the TXM) and overall state (no obvious grid surface or cell sample disruption). Samples were then imaged in both green and red fluorescence to identify individual cells within the population that both expressed fluorescent Gal3 and were infected with fluorescent virus. 3D-SIM data were collected on a number of these representative cells (4 mock-infected controls, 8 at 1h after infection, 11 at 2 h after infection, 11 at 3 h after infection and 5 at 4 h after infection). Data were reconstructed with SoftWoRX 6.5.2 (GE Healthcare) using real optical transfer functions generated from 3D-SIM images of 175 nm single-color fluorescent beads (PS-Speck, Thermo Fisher Scientific) to produce super-resolution image stacks. Multi-channel images were aligned with Chromagnon (Matsuda et al., 2018). The raw and reconstructed data were analyzed in Fiji (Schindelin et al., 2012) using SIMcheck (Ball et al., 2015) to ensure the results were realistic and contained no artifacts.

#### Cryo-soft X-ray Tomography and X-ray data reconstruction

X-ray data were collected with an UltraXRM-S/L220c X-ray microscope (Carl Zeiss X-ray Microscopy, Inc.) at beamline B24 (DLS) using 500 eV X-rays. This instrument is fitted with a capillary condenser, a 40nm zone plate objective (25 nm for the BSC-1 work) and a 1024B Pixis CCD camera (Princeton instruments). Samples were loaded into the microscope chamber in batches of four and were assessed for structural integrity and alignment potential, inspecting them first with the in-line visible light 20x objective to give an overall map of the grid using visible light (images recorded on a Retiga 4000R camera; Teledyne). X-rays were then used to generate X-ray 2D mosaic maps of grid boxes that contained ROIs. The visible light microscopy setup benefits from a variable visible light LED which was used to confirm fluorophore presence and agreement with the fluorescence signal recorded in the cryoSIM. Tilt series were collected from –65° to +65° at increments of 0.5° on 3 mock-infected cells, 4 cells at 1h after infection, 8 cells at 2 h after infection, 4 cells at 3h after infection and 5 cells at 4h after infection (all previously imaged at the cryoSIM). All data were aligned and reconstructed automatically to tomograms using the in-house pipeline which employs Batchruntomo (Mastronarde and Held, 2017) via a number of scripts to allow parallel and near real-time processing. Specifically, three protocols for alignment and reconstruction are currently provided: weighted back projection (WBP), simultaneous iterative reconstruction techniques (SIRT) and automatic patch tracking. Batchruntomo (Mastronarde and Held, 2017) is part of the IMOD package (version 4.9.2) (Kremer et al., 1996). Where necessary, multiple adjacent sites were used for data collection for subsequent stitching and FOV expansion.

#### Correlative cryo-SXT / cryo-SIM imaging

Equivalent cryo-SIM and cryo-SXT datasets were overlaid with the multidimensional registration software eC-CLEM (Paul-Gilloteaux et al., 2017) (Figure S4). Initially, cryo-SXT tomograms and chromatic drift-corrected multichannel SIM images were relocated. For this the 2D dimensional transformation between SIM data and X-ray tomograms was computed using the 2D rigid registration mode of eC-CLEM (coarse alignment) (Paul-Gilloteaux et al., 2017). This transformation was computed as a combination of 2D independent paired relocations; (a) SIM data on brightfield image stacks, (b) brightfield data on 2D X-ray mosaics and (c) X-ray mosaics on X-ray tomogram positions. Note that accuracy is not the main target in this step, in particular because volumes were processed as 2D data. The 2D transformation from SIM to X-ray tomogram was then applied to align in 2D the 3D SIM and X-ray ROI data, as initialisation for the complete 3D alignment step. Features used for alignment purposes included both the nanoparticles added on samples just before vitrification as well as grid patterns (grid bars, carbon substrate holes and sample imperfections) and cellular features that displayed good contrast in both X-ray and fluorescence microscopy (fluorescent endosomes in this case). The next phase involved the accurate 3D registration of the 3D chromatic drift-corrected cryo-SIM data to the respective 3D cryo-SXT data, using the 3D rigid mode of eC-CLEM (Paul-Gilloteaux et al., 2017). We note that no deformation was used to align data; the only allowed degrees of freedom in this process were 3D rotation, translation and isotropic scaling of data (which was found to be negligible at about 2%).

### Quantification and Statistical Analysis

In Figure 6C, error bars indicate standard deviation of n = 8 in b and n = 9 in (B) and (C).
