# Supplementary material for: 3D Correlative Cryo-Structured Illumination Fluorescence and Soft X-ray Microscopy Elucidates Reovirus Intracellular Release Pathway
Source: Cell. 2020 Jul 23;182(2):515–530.e17. doi: 10.1016/j.cell.2020.05.051 (PMC7391008; doi:10.1016/j.cell.2020.05.051)
Supplement: Document S1. Tables S1–S2 [file mmc1.pdf]

Cell, Volume *182*

Supplemental Information

3D Correlative Cryo-Structured Illumination Fluorescence and Soft X-ray Microscopy Elucidates Reovirus Intracellular Release Pathway

Ilias Kounatidis, Megan L. Stanifer, Michael A. Phillips, Perrine Paul-Gilloteaux, Xavier Heiligenstein, Hongchang Wang, Chidinma A. Okolo, Thomas M. Fish, Matthew C. Spink, David I. Stuart, Ilan Davis, Steeve Boulant, Jonathan M. Grimes, Ian M. Dobbie, and Maria Harkiolaki

Supplemental Table 1. Optical resolution values for representative structured illumination data at beamline B24. Related to Figure 1.

**Wavelength Ex/Em (nm)**	**Theoretical WF XY resolution**	**Measured WF XY resolution**	**Theoretical SIM XY resolution**	**Measured SIM XY resolution**	**Theoretical Z resolution**	**Measured WF Z resolution**	**Measured SIM Z resolution**
405/452	310	360	160	240	680	790	540
488/525	360	420	190	200	790	930	520
561/605	410	440	210	220	910	1000	590
647/680	460	580	240	320	1020	1510	850

Resolution parameters for the cryoSIM in nm for the lateral (XY) and axial (Z) directions at the 4 wavelengths available giving theoretical resolutions for widefield images and measured FWHM values from fitted Gaussians for widefield and SIM images in the lateral and axial directions. Theoretical resolutions are derived from the Rayleigh Criterion, $d = 1.22\ \lambda / 2NA$ for the lateral measures and $d = 1.22\ \lambda / NA^2$ for axial, where λ is the emission wavelength and the objective NA is 0.9. Theoretical SIM resolutions were calculated from $1/d = (1/r) + (1/s)$, where d is the achievable resolution, r is the widefield resolution at that wavelength and s is the illumination stripe width used for that wavelength.

Supplementary Table2. Optical performance values for the transmission X-ray microscope water-window imaging. Related to Figure 2.

Higher resolution dr_N = 25 nm

Condenser $NA_{2.4nm}$=0.031 (31 mrad) ; Objective $NA_{2.4nm}$=0.0477 (47.7 mrad)
TXM optical system presents as partially coherent (NA condenser/NA objective <1)

<u>**Raw Data**</u>

Magnification=1300
FOV=10 nm
Pixel size=10 nm

DOF=1µm
Depth/Axial resolution=0.5 µm

Lateral resolution
Theoretical/Expected Rayleigh = 30.5 nm
Theoretical/Expected (partial coherence) = <15nm (leveraged against loss of contrast)

Measured (Siemens star) = 120 nm (signal: noise=5:1; Rose criterion compliant)
60 nm (signal: noise=4:1; 3σ compliant)
30 nm (signal:noise=2:1)

<u>**Reconstruction**</u>
Expected $lateral_{tilt\ series}$ = @ 0.5 µm: <40 nm (up to θ +/-65°)
@ 5.0 µm: <40 nm (up to θ +/-2.8°)

$FSC_{raw\ data(tilt\ series)}$ 3σ = 90 nm
½ bit = 115 nm
$FSC_{reconstructed\ data}$ 3σ = 94 nm
½ bit = 120 nm

Lower resolution dr_N = 40nm

Condenser $NA_{2.4nm}$=0.031 (31 mrad) ; Objective $NA_{2.4nm}$=0.0298 (29.8 mrad)
Optical system presents as almost fully incoherent (NA condenser/NA objective ≈ 1)

<u>**Raw data**</u>

Magnification=812.5
FOV=16 nm
Pixel size=16 nm

DOF=2.6 µm
Depth/Axial resolution=1.3 µm

Lateral resolution
Theoretical/Expected (no coherence) = 48.8 nm
Theoretical/Expected (partial coherence) = up to 24 nm (leveraged against loss of contrast)
Measured (Siemens star) = 120 nm (signal: noise=5:1; Rose criterion compliant)
60 nm (signal: noise=3:1; 3σ compliant)
30 nm (signal: noise=1.2:1)
<u>**Reconstruction**</u>

$Expected_{tilt\ series}$ =	@ 0.5 µm:<br>@ 5.0 µm:	<60 nm (up to θ +/-70°)<br><60 nm up to θ +/18°)
$FSC_{raw\ data\ (tilt\ series)}$	3σ = 127 nm<br>½ bit = 145 nm	
$FSC_{reconstructed\ data}$	3σ = 155 nm<br>½ bit = 183 nm	

Theoretical resolutions for incoherent illumination are derived from the Rayleigh Criterion, $d= 1.22\ \lambda/ 2NA$ for the lateral measures and $d= 1.22\ \lambda/NA^2$ for axial, where λ is 2.5 nm (500 eV). Magnification was calculated as m=physical pixel size/ recorded pixel size. All values of theoretical resolutions for partially-coherent imaging are estimated based on doubling of resolution as the optical transfer function approaches zero on the caveat that useful contrast will have been lost before such value is reached. All measured values have been assessed with λ=2.5 nm (working illumination wavelength at beamline B24 is 2.5 nm or 500 eV). Fourier Shell Correlation estimates were generated from representative X-ray data (U2OS cells) using the EBI Fourier Shell Correlation server at: https://www.ebi.ac.uk/pdbe/emdb/validation/fsc/ (van Heel and Schatz, 2005).
